# Declines of ebony and ivory are inextricably linked in an African rainforest

**DOI:** 10.1126/sciadv.ady4392

**Published:** 2025-08-27

**Authors:** Vincent Deblauwe, Matthew Scott Luskin, Serge Désiré Assola, Olivier J. Hardy, Simon Jansen, Céline Loubières, Gaston Guy Mempong, Jean Mathurin Ntsihe, Gilbert Oum Ndjock, Eric Rostand Onguene Kwecheu, Luke L. Powell, Bonaventure Sonké, Thomas B. Smith

**Affiliations:** ^1^ International Institute of Tropical Agriculture, Yaoundé, Cameroon.; ^2^ Center for Tropical Research, Institute of the Environment and Sustainability, University of California, Los Angeles, Los Angeles, CA, USA.; ^3^ Congo Basin Institute, University of California, Los Angeles, Los Angeles, CA, USA.; ^4^ School of the Environment, University of Queensland, Brisbane, Australia.; ^5^ Centre for Biodiversity and Conservation Science, University of Queensland, Brisbane, Australia.; ^6^ Evolutionary Biology and Ecology Unit, Faculty of Sciences, Université Libre de Bruxelles, Brussels, Belgium.; ^7^ Institute of Silviculture, Department of Forest and Soil Sciences, University of Natural Resources and Life Sciences (BOKU), Vienna, Austria.; ^8^ Evolutionary Biology and Ecology Unit, Faculté des Sciences, Université Libre de Bruxelles, Avenue F. D. Roosevelt 50, CP 160/12, B-1050, Brussels, Belgium.; ^9^ Ministry of Forestry and Wildlife, Yaoundé, Cameroon.; ^10^ CIBIO-InBIO, Research Centre in Biodiversity and Genetic Resources, University of Porto, Vairão, Portugal.; ^11^ BIOPOLIS Program in Genomics, Biodiversity, and Land Planning, CIBIO, Vairão, Portugal.; ^12^ Biodiversity Initiative, Houghton, MI, USA.; ^13^ Institute of Biodiversity, Animal Health and Comparative Medicine, University of Glasgow, Glasgow, UK.; ^14^ Plant Systematics and Ecology Laboratory, Department of Biology, Higher Teachers’ Training College, University of Yaoundé I, Yaoundé, Cameroon.; ^15^ Department of Ecology and Evolutionary Biology, University of California, Los Angeles, Los Angeles, CA, USA.

## Abstract

Critically endangered African forest elephants preferentially eat fruits and disperse seeds of carbon-dense trees, including the highly valued and threatened African ebony. The illegal ivory trade has led to severe declines in elephant populations, but the long-term impacts on tree species are poorly understood. Using a comprehensive dataset including age-class, spatial, genetic, and experimental data, across a hunting pressure gradient, we show how paired declines in elephant and ebony populations are linked by a previously unrecognized mutualism in which elephant dung protects ebony seeds against seed predators. Disruption of this mutualism by poaching exacerbates seed predation by herbivores and was associated with a 68% reduction in small sapling recruitment. This threat to the survival of a valuable and iconic tree species raises concerns about the far-reaching consequences of forest elephant extermination.

## Teaser

Loss of critically endangered forest elephants reduce the regeneration of a threatened species of ebony tree.

## INTRODUCTION

Unlike Southeast Asia and the Neotropics, African tropical forests retain their prehistoric megafauna (*1*) and the associated long-distance seed dispersal function (*2*). However, this distinction is disappearing because African forest elephants (*Loxodonta cyclotis*), hereafter referred to as “elephants,” are poached for ivory, leading to an 86% population decline in the past three decades and an extremely high risk of extinction in the near future (*3*). Elephants are ecosystem engineers that help create and maintain forest habitats by recycling and distributing nutrients, clearing the understory, browsing, and dispersing seeds (*4*–*7*). They are selective browsers (*8*) that tend to limit fast-growing, low wood-density trees through preferential trampling, debarking, and herbivory while dispersing seeds of large, carbon-dense trees (*9*). The collapse of elephant populations is associated with a reduced seed dispersal distance of the so-called “megafaunal-syndrome” trees that are deemed to primarily rely on elephants for the dispersal of their seeds (*10*, *11*).

It follows that populations of elephant-dispersed trees are expected to decline jointly with elephants (*6*, *12*). The shift toward trees of reduced size and wood density, together with an altered forest composition and structure, is estimated to have caused a reduction in the equilibrium aboveground biomass of central African forests of ~7% (*13*). Recent declines in small sapling abundance of some elephant-dispersed trees have been reported (*10*, *14*), but the scale and magnitude remain unclear (*15*). The prevailing view is that losing elephant gut passage and long-distance seed dispersal will be detrimental to regeneration. Some seeds have higher germination and growth rates after passing through animal digestive tracts (*16*, *17*). The Janzen-Connell hypothesis proposes that host-specific enemies maintain tropical forest diversity by creating a relatively inhospitable area near conspecifics (*18*, *19*). Dispersal can provide an escape from specialized enemies and intraspecific competition, reducing mortality (*20*–*22*). However, neither the benefits of elephant gut passage nor the escape from density-dependent mortality are consistent across tree species (*11*, *23*, *24*). Last, it has been hypothesized that the postdispersal predation rate in elephant dung could be lower than predispersal predation under the parent tree (*25*). However, postdispersal predation of small seeds can be very high in elephant (*26*) or primate (*27*) dung, and pigs and antelopes (Cephalophinae) are known to regularly eat large seeds in elephant dung (*28*).

Here, we examine whether the severe population declines of elephants in recent decades are associated with reduced regeneration of African ebony (*Diospyros crassiflora* Hiern, hereafter “ebony”) and test the ecological underpinnings of the elephant-ebony mutualism. Ebony is a shade-tolerant tree that grows up to 25 m in height, with a trunk diameter of up to 1.2 m. It bears large fleshy fruits of 10 cm by 6.5 cm, containing up to 10 large seeds of 5 cm by 2 cm by 1.5 cm. Ebony is among the slowest-growing species in the Congo Basin, with a diameter increment of ~1.7 mm year^−1^ in undisturbed forests [(*29*) and references therein]. The species is dioecious; only half the population produces pistillate (female) flowers and fruits. Its population faces major threats from deforestation (*30*) and overexploitation for its high-value jet-black wood (*29*). Its fruits are a preferred food source for elephants, and seeds are commonly found in their dung (*31*, *32*), as confirmed by our observations. The fruits exhibit traits that have been associated with a megafaunal syndrome: large, fleshy fruits (>4 cm in diameter); large seeds (>2 cm); brown, green, or yellow coloration; strong odor (*11*, *33*, *34*); and a distinctive noise when falling and striking the ground at maturity (*14*). Gorillas and chimpanzees also consume ebony fruit (*29*). Still, they likely act as seed predators, although gorillas may disperse a small proportion of ingested seeds, as reported for *Diospyros mannii* (*35*), a related species with similar fruits and seeds. Other primates cannot swallow such large seeds or carry entire ebony fruits in their cheek pouches. However, fruits could be dispersed by stomatochory, wherein the disperser carries fruits and removes the flesh at distances typically under 150 m for monkeys (*36*, *37*). While stomatochoric dispersal of fruits of this size is plausible for monkeys, it is unlikely for other taxa, including apes (*36*). Canopy dispersal of ebony fruit is also doubtful, as we observed that fruits mature only after falling to the ground, one of the characteristic traits of elephant obligate fruits (*38*). Antelopes have been reported to consume ebony fruit (*29*). Still, our recent investigation of 78 nests and 124 dung piles during and after the peak of ebony fruit fall revealed no ebony seeds (*39*). This suggests that antelopes crush seeds during rumination, a common outcome for large, soft seeds (*40*).

We hypothesize that ebony is elephant-dependent for its dispersal and that removal of megafauna in hunted forests has increased sapling clumping. The life history traits of ebony—shade tolerance, small height, and large seeds—are associated with weaker conspecific negative density dependence (CNDD) in other systems (*23*, *24*, *41*, *42*). To investigate this, we experimentally estimated the strength of CNDD to understand whether dispersal limitation is the driver of increasing mortality in defaunated forests or whether other mechanisms are at play, such as increased seed predation by rodents.

We combined field observations of ebony population demography, parentage analysis, and experimental trials to address the following questions and hypotheses. We start by asking whether the loss of elephants is associated with observable effects on the dynamics of the ebony population. We hypothesized that sites with fewer elephants would be associated with lower ebony dispersal distances, lower recruitment, and increased spatial genetic structure. Furthermore, to explain the cause of these effects, our next step was to examine whether dispersal distance influenced ebony recruitment, as recent work has questioned the importance of this mechanism for tropical trees (*23*). We tested the classic hypothesis that seed and seedling mortality would increase with the density of conspecifics. Last, we asked whether the primary dispersal mode affects seed fate. We tested whether dung protects ebony seeds from predation and whether ebony seed passage through elephant gut increases germination success.

## RESULTS AND DISCUSSION

**Fig. 1.**
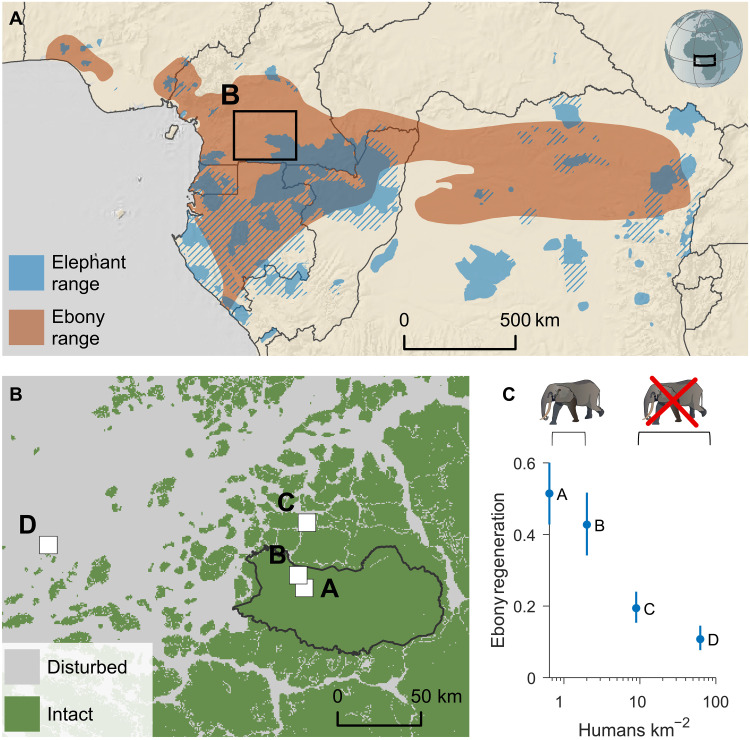
Study location and context. (**A**) Current range of forest elephant (solid known, striped possible) (*86*). Elephants occupied the entire Congo Basin in the early 19th century before modern ivory trade and now overlap with a third of ebony historical range [adapted from (*29*); https://creativecommons.org/licenses/by/4.0/]. (**B**) Locations of the four study sites in the humid tropical forest of southeastern Cameroon relative to the Dja Faunal Reserve (black boundary). The reserve shelters a forest with high ecological integrity (green area) (*87*). Elephant abundance has decreased down to 0.04 km^−2^ in 2018 in sites A and B (*66*), both located >7 km from human settlements and well within the Dja Reserve. Hunting pressure is comparatively high in sites C and D, where elephants, apes, monkeys, and large duikers have largely been extirpated (*56*, *73*, *76*). (**C**) Ebony regeneration, expressed as the ratio of juveniles over adults, is inversely proportional to the density of the human population in the area. More densely populated site D is expected to have lost its megafauna earlier than site C.

We sampled ebony in 4-km^2^ plots in four unlogged forests in Cameroon that span a gradient of hunting pressure (Fig. 1 and fig. S1). All ebony trees at least 130-cm tall were inventoried and classified into three diameter cohorts based on diameter at breast height (dbh; stem diameter measured at 130 cm above ground): adults (dbh of ≥10 cm); smaller [i.e., juvenile (fig. S2) trees divided into large saplings (5 cm ≤ dbh < 10 cm)], and small saplings (dbh of <5 cm). The spatial distribution of saplings was calculated using individual geographic coordinates, and long-term regeneration dynamics were inferred from dbh distributions. Seed dispersal distances were inferred by assessing allelic diversity and parentage of inventoried individuals. To examine the relative importance of different mechanisms through which elephant frugivory may benefit ebony seeds, we experimentally manipulated in situ seed dispersal distances from mother trees in two sites (sites A and C, Fig. 1B) and seed substrate in one site (site A, Fig. 1B).

### Ebony demography under low and high hunting pressure

**Fig. 2.**
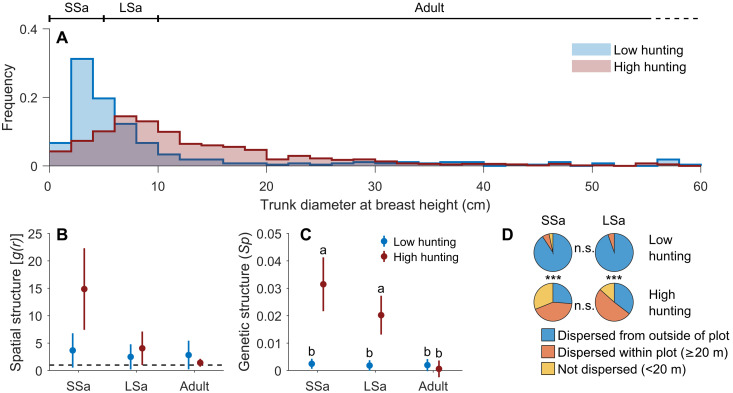
Ebony population parameters of saplings and adults in different hunting regimes. (**A**) Size-class distribution of ebony in sites with low versus high hunting pressure, standardized by the number of censused trees per site. The histogram shows dbh in 2-cm bins, and the lack of ebony in the 0- to 6-cm size is the “missing cohort” in high-hunting regimes. Abscissa is truncated at 60 for clarity. (**B**) Inhomogeneous pair correlation function (PCF) and 95% CI at 10 m distance, *g* (*r* = 10), shows the amplitude of clustering of each cohort relative to complete spatial randomness (*g* = 1). (**C**) Magnitude and 95% CI of fine-scale spatial genetic structure (FSGS) of each cohort expressed by the *Sp* statistic calculated from the rate of decrease of kinship coefficients between individuals as a function of ln(*Distance*). Letters are for statistically significant differences (two-tailed paired-sample *t* test with Holm-Bonferroni correction: *P* ≤ 0.05). (**D**) Association between hunting pressure and dispersal distance for saplings. Distances (*D*) from identified mother as obtained from analysis of parentage is classified as follows: not dispersed (*D* < 20 m), dispersed within plot (*D* > 20 m and mother identified), and dispersed from outside of plot (mother not identified). Statistical significance indicated as *P ≤ 0.05, ***P ≤ 0.001, and n.s. for not significant or *P* > 0.05 (Fisher’s exact test with Holm-Bonferroni correction). Whiskers for 95% CI. LSa, large sapling; SSa, small sapling.

To understand the impact of wildlife extirpation on regeneration, we examined evidence based on demography. We first quantified the regeneration status of ebony populations in elephant-present and elephant-absent forests. Ebony tree size-class distributions significantly differed between elephant-present and elephant-absent forests [two-tailed two-sample Kolmogorov-Smirnov (K-S) test, *D* = 0.370, *P* < 0.0001; Fig. 2C and fig. S3]. Small saplings (dbh of <5 cm) accounted for 47.2% of all individuals in forests with elephants but just 15.1% where elephants were absent [Fisher’s exact test, odds ratio (OR): 5.05, 95% confidence interval (CI): 3.67 to 6.93, *P* < 0.0001; Fig. 1C]. This equates to 68.1% (CI: 60.3 to 74.4%) lower small-sapling prevalence in forests without elephants compared to those with elephants, strongly in support of the hypothesis that elephant defaunation is driving the co-collapse of ebony. In forests with low hunting pressure, the loss of regeneration started more recently, as indicated by a drop in frequency in the 0- to 2-cm dbh class, which coincides with the documented decline of elephant abundance in the two decades preceding our inventory (*33*). Loss of regeneration in the high-hunting regime was visible in the dramatic suppression of the cohort of trees between 0 and 6 cm in dbh relative to larger trees of prior decades (Fig. 2A).

If the loss of ebony regeneration under a high-hunting regime is due to the extermination of elephant seed dispersers, we would expect a reduction in dispersal distance revealed by increased clustering of ebony progeny. The pair correlation function (PCF) represents how the probability of finding a pair of points at a certain distance compares to what would be expected under a random (Poisson) distribution. Clustering (two-tailed Monte Carlo test, α = 0.05; fig. S4) occurred at small distances between small saplings in each of the four sites, with a significant correlation at up to ~50 m. This distance is consistent with the cluster size of gravity-dispersed trees observed in other humid tropical forests (*43*). At a 10-m radius, i.e., roughly the radius of large adult tree crowns, the amplitude of clustering under low-hunting conditions remained relatively constant across cohorts, with a PCF 1.3 times higher for small saplings compared to adults. In high-hunting regimes, however, clustering was 10.5 times higher in small saplings (Fig. 2B), suggesting either higher CNDD in the high-hunting regimes or a progressive reduction in dispersal distance in recent decades.

To draw inferences regarding dispersal distance that are unbiased by the possible existence of distance-dependent mortality, we quantified the fine-scale spatial genetic structure (FSGS) in the two hunting regimes from the decay of the kinship coefficient, *F*, between trees at increasing distance (kinship-distance curve) (*44*). We found that the average relatedness of small ebony saplings separated by less than 30 m in a high-hunting regime (*F* = 0.155) was nearly double that in low-hunting regime (*F* = 0.085; fig. S5). This former value indicates the presence at short distances of a mixture of half-siblings (*F* = 1/8) and full siblings sharing both parents (*F* = 1/4).

The extent of FSGS, i.e., the decrease of kinship between individuals as spatial distance increases, was quantified and compared among populations using the *Sp* statistic derived from the kinship function. Higher *Sp* is expected when either gene dispersal distance or population density decreases (*44*). Despite the lower abundance of ebony in forests with low hunting pressure, the *Sp* value for small saplings was lower (0.0025) than in high hunting pressure (0.0314; two-tailed paired *t* test, *t*(16) = 5.88, *P* = 2.33 × 10^−5^; Fig. 2C). This is biologically equivalent to shifting from a plant being animal-dispersed ( $\bar{\mathrm{Sp}}$ = 0.0088) to being gravity-dispersed ( $\bar{\mathrm{Sp}}$ = 0.0281) (*44*, *45*). In elephant-absent forests, spatial genetic structure decreased with age of individuals, from small saplings (*Sp* = 0.0314) to large saplings (*Sp* = 0.0202) to adults (*Sp* = 0.0018; Fig. 2C), in support of a progressive loss of seed dispersal function over recent decades. By contrast, in forests with elephants, *Sp* remained low and constant between age cohorts (*Sp* = 0.0025, 0.0018, and 0.0020; two-tailed paired *t* test), suggesting the maintenance of seed dispersal function by elephants.

A population’s spatial genetic structure depends on both seed and pollen dispersal. To disentangle the contribution of each, we relied on parentage analyses to infer past dispersal events and on the neighborhood model to estimate dispersal kernels and rates of immigration into the 4-km^2^ sampling plots for seeds and pollen. The results show that the seed immigration rate in the low-hunting site was five times higher than in the high-hunting area, as estimated by the proportion of juveniles whose maternal parent was not found inside the inventoried plots [84% in sites A and B (CI: 79 to 90%) versus 16% in sites C and D (CI: 12 to 21%)]. The same pattern is observed in the individuals’ parentages (Fig. 2D). The proportion of undispersed small saplings (within 20 m of their mother) was 3% in the low-hunting regime compared to 31% in the high-hunting regime. These results suggest that vertebrates able to disperse seeds at small distances, such as scatter-hoarding rodents or stomatochoric monkeys, make a negligible contribution to both primary seed dispersal and effective regeneration of ebony when hunting pressure is low. Given the size of our forest inventories (2 km by 2 km), the dominance of immigrant small saplings in forests with low hunting pressure is consistent with the estimation of 89% of seeds dispersed farther than 1 km by the forest elephant (*46*).

In forests with high hunting pressure, seed dispersal efficiency decayed progressively in the past decades, as shown by the mean distance between identified mothers and their progeny, which is 460 m (CI: 337 to 584 m) for large saplings (50 to 100 years old) and 206 m (CI: 80 to 332 m) for small saplings (<50 years old). This latter distance is compatible with dispersal by stomatochoric monkeys (*36*) or scatter-hoarding rodents (*47*), thus suggesting the progressive increase in relative contribution by these organisms.

Pollen dispersal appeared more limited than seed dispersal, with mean dispersal distances estimated at 453 m (CI: 351 to 636 m) in the low-hunting forest and 258 m (CI: 228 to 296 m) in the high-hunting forest. The shorter distance in the hunted forest is explained by the higher density of adult trees in site C, making *D. crassiflora* one of the few plant species for which seed dispersal naturally exceeds pollen dispersal (*45*, *48*–*50*). These distances are comparable to those reported for other tree species dispersed by small insects (*45*) like the hymenopterans that pollinate ebony (*51*). Despite lower population density and longer pollen dispersal distance in the low-hunting area, the pollen immigration rate was only half that of the high-hunting site, as estimated by the proportion of juveniles whose father was not found inside the inventoried plots [21% in sites A and B (CI: 9 to 34%) versus 41% in sites C and D (CI: 35 to 47%); table S4]. This pattern likely reflects a greater contribution to regeneration by well-connected mother trees in low-density areas, who receive mostly local pollen, while more spatially or phenologically isolated mothers relying on external pollen sources have lower fecundity, lowering the overall immigration rate.

### Negative density dependence

We used experimental trials to test whether higher sapling clustering where elephants are extirpated produces ecologically meaningful consequences. We conducted a seed dispersal manipulation experiment to evaluate whether ebony benefits from dispersal away from conspecific trees, which act as specific enemy reservoirs (also known as the Janzen-Connell hypothesis). We collected and placed seeds on the ground in two transects radiating from the target conspecific adult tree in opposite directions. At 2, 20, and 50 m from the trunk of the adult tree, seeds were sown 20 cm apart in subplots at densities of either 2 or 20 seeds depending on the transect (see table S2 for sample sizes). We repeated the experiment in forests under low and high hunting pressure and assessed seed survival at 2 months and seedling survival at 24 months.

**Fig. 3.**
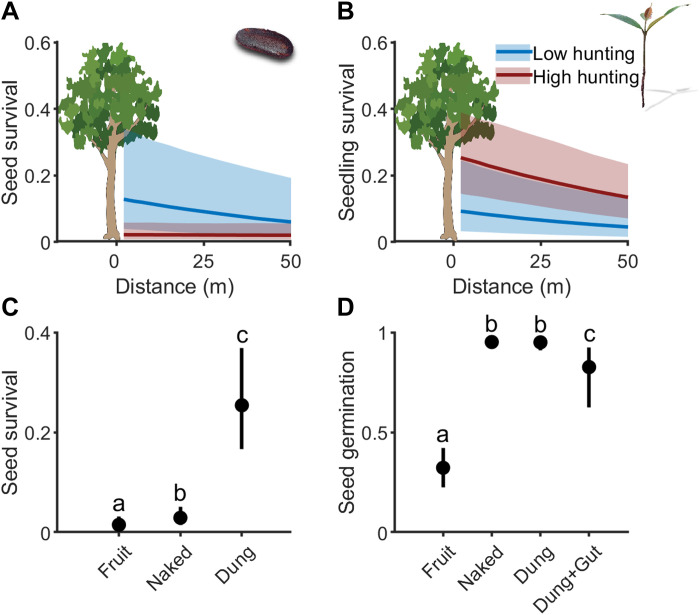
Effect of elephants on postdispersal seed fate. Seed (**A**) and seedling (**B**) survival rate at 2 months postsowing and 2 years, respectively, as a function of distance to the nearest adult and hunting pressure. Seed survival in hunted forests is lower due to rodent predation, and seedling survival is lower in low-hunting forests due to higher herbivory by antelopes. (**C**) Seed survival against mammal predation risk at 2 months postsowing (*n* = 550 for fruit, naked, and dung treatments) compared between undispersed (fruit), naked, and dung environment. (**D**) Seed germination rate in exclusion cages (*n* = 300 for each treatment and *n* = 44 in gut) compared among undispersed (fruit), naked, and dung with and without elephant gut passage. Letters are for statistically significant differences (Tukey’s test: *P* ≤ 0.05). Sample sizes and details for all graphs are provided in tables S5 and S6. Shown are predicted results from best-fit models and 95% CIs.

The best-fit model for seed survival at 2 months after experimental dispersal showed that both distance to conspecific adults and hunting pressure negatively influenced seed survival, with a significant interaction between these variables [likelihood ratio test (LRT) of model with interaction versus additive effects only: *P* = 0.0105, χ*^2^* = 6.55, df = 1; LRT versus null model: *P* < 0.001, χ^2^ = 18.798, df = 3; Fig. 3A and table S6]. The CIs overlapped with the constant model, so the negative relationship between distance and survival of seeds, opposite to Janzen-Connell’s, was weakly supported. Models that included distance significantly outperformed the null model via LRTs (*P* < 0.0001; table S6). Although exceptions have been reported (*52*), increased removal of seeds by rodents under high hunting pressure is consistent with patterns observed in other tree species in the Congo Basin (*53*) and is usually attributed to the proliferation of small-bodied mammals (*54*, *55*). This interpretation is further supported by our camera trap observations, which recorded a higher rate of seed removal by rodents in the high-hunting regime (fig. S6), as well as by Pavan’s report (*56*) of increased rodent abundance at this site. Seed density on the ground had no effect on germination (LRT: *P* = 0.93, χ*^2^* = 0.0075, df = 1). This finding is consistent with a recent synthesis finding high variability in the strength of Janzen-Connell effects among tropical tree seeds (*23*).

The best-fit model for seedling survival to 24 months showed a negative effect of distance to conspecific adults, also opposite to the Janzen-Connell hypothesis, but weakly supported due to large uncertainty (LRT of best-fit model versus the null model: *P* < 0.0001, χ^2^ = 25.4, df = 2; Fig. 3B and table S6). Models that included distance significantly outperformed the null model via LRTs (*P* < 0.0001; table S6). Overall, we found no strong evidence that the survival of seeds or seedlings varied with distance from conspecifics. This suggests that a reduction in dispersal distance alone is not expected to strongly affect ebony tree regeneration within 2 years postgermination. This is consistent with findings outside Africa, where smaller- and larger-seeded trees experience weaker CNDD (*24*, *57*). Camera traps revealed that the lower survival in the low-hunting forest was due to the predation of 20% of seedlings by forest antelopes that were virtually absent from forests under high hunting pressure (Fisher’s exact test, OR: 0, *P* = 1.52 × 10^−15^; fig. S6).

### Postdispersal seed fate

Since rodents are abundant seed predators irrespective of hunting pressure, we investigated whether elephants provide protection against seed predation by rodents. Our best-fit model of seed survival at 2 months showed that elephant dung provided a strong protective effect against rodent seed predation (LRT versus null model: *P* < 0.0001, χ*^2^* = 259.45, df = 2). Specifically, naked seeds were 8.5 times more likely to be predated than those within elephant dung (CI: 4.2 to 22.2, Tukey’s test: *P* < 0.0001) and 15.6 times more likely than seeds remaining within fruits (CI: 5.6 to 44.0, Tukey’s test: *P* < 0.0001; Fig. 3C). In all treatments exposed to mammal predation, cameras revealed that the predation/removal were primarily by Emin’s pouched rats (*Cricetomys emini*) and occasionally by yellow-backed duikers (*Cephalophus silvicultor*; fig. S7).

Our best-fit model for seeds experimentally protected from mammals within cages showed that seeds in elephant dung were three times more likely to germinate after 2 months than seeds left inside fruits (Tukey’s test: *P* < 0.0001; *r^2^* = 0.322; LRT: *P* < 0.001, χ^2^ = 371.35, df = 3; Fig. 3D). This positive effect of dispersal can be explained by the removal of pulp because seeds in dung and naked seeds on the ground had similar germination rates (95.2 and 95.0%, respectively; Tukey’s test: *P* = 0.998). The high germination and mortality rates of seeds within two months indicate that they do not exhibit dormancy and do not survive in the soil seed bank. Gut passage decreased germination rate by 12.3% when comparing seeds found in natural dung with those experimentally placed in dung (without gut passage; Tukey’s test with sequential Bonferroni correction: *P* = 0.0298). Seed predation by insects or fungi did not vary between naked and dung treatments and was negligible, given the relatively high germination rate. Dung provides strong net positive effects regardless of whether the seed was naturally digested or experimentally moved, and the effect is an order of magnitude larger than the increased seed predation we observed in highly hunted forests. This means that the loss of dung protective effects from rodent seed predators in hunted forests may entirely explain the decline in ebony regeneration.

### Implications

**Fig. 4.**
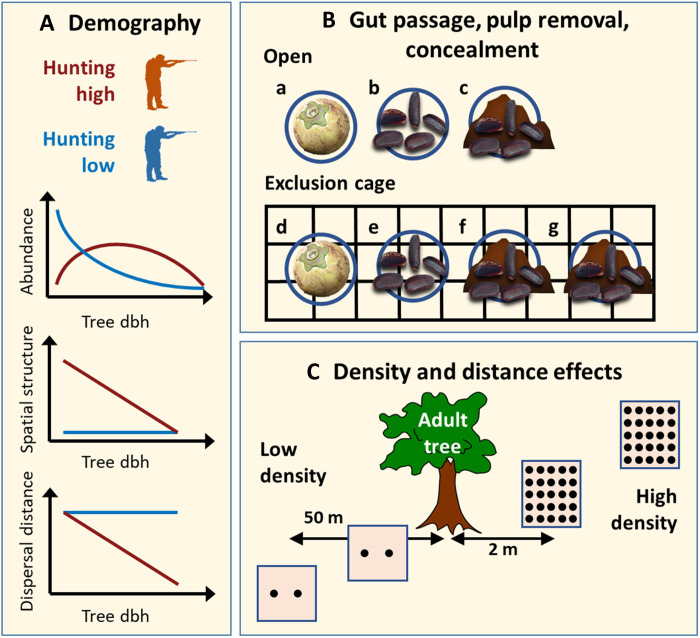
Hypothesized relationships between elephants, ebony, and hunting. We combined field observations of ebony population demography (**A**), and experimental trials (**B** and **C**) to evaluate our hypothesis. (A) We asked whether the loss of elephants is associated with observable ebony population dynamics effects and found a decline in regeneration and seed dispersal distance in hunted forests. (B) To explain the cause of these effects, we tested whether seed passage in elephant gut increases germination success and whether dung protects seeds from predation. Germination was not affected by seed passage through the elephant gut [(f) versus (g)], but pulp removal increased germination success [(d) versus (e) and (f)]. Dung reduced seed predation by rodents [(c) versus (a) and (b)]. (C) We tested the hypothesis that seed and seedling mortality would increase with the density or distance of conspecifics (Janzen-Connell hypothesis) and found no positive association.

This study systematically and experimentally investigates the fate of an elephant-dispersed tree species following the removal of its disperser, as well as the mechanisms involved (Fig. 4). We find that a decline in elephants is associated with ebony trees losing both seed dispersal and small sapling recruitment potential. The drop in juvenile recruitment (dbh of <10 cm) in hunted areas is consistent with the recorded extirpation of elephants from these sites a few decades ago given the growth rate of ebony. This negative consequence is therefore likely overlooked by many standard forest inventory datasets, where only trees ≥10 cm are recorded (*58*). We found evidence that elephants provide a nonredundant seed-dispersal service. Smaller dispersers, such as scatter-hoarding rodents and stomatochoric monkeys, did not contribute substantially to effective seed dispersal in the low-hunting forests and could not sustain species regeneration at levels achieved by elephants. These findings support our hypothesis that ebony dispersal is elephant dependent. The net positive effect of elephant presence on ebony tree regeneration suggests that the benefit of dung on seed survival and germination has offset the higher seedling herbivory in low-hunting regimes. In our study, we did not investigate the possible decrease in trampling associated with elephant loss (*7*, *13*), but if present, this effect is also offset.

Our results on the outsized positive role of dung challenge the notion that seed dispersal benefits primarily arise from spatial movement. Instead, they indicate that dung confers direct benefits to ebony seeds by concealing them from predators. This previously underappreciated mechanism highlights gaps in our understanding of the role of seed dispersal in forest dynamics and the maintenance of biodiversity. Further investigation into postdispersal seed fate and the interplay between dispersers and predators competing for the same seeds is needed. Our results raise concerns about the future of elephant-dependent species if concealment from seed predators is a key function provided by elephant manure. Fruits are an essential resource for forest elephants (*8*, *59*), and investigating whether obligate fruits form a substantial part of their preferred diet is crucial. Disruption of this potential positive feedback loop (*60*) could ultimately render Congo Basin forests devoid of elephants and unsuitable for their return due to diminished food sources.

We found rotting ebony fruits on the ground where elephants are absent, which our results indicate are far less likely to survive and germinate than dispersed seeds (fig. S8). Some of the seeds eventually germinate, however, forming a carpet of seedlings below the crown of healthy female adults. We found no evidence that these suffer higher mortality in the first 2 years than dispersed plants. It remains unclear whether intracohort competition will ultimately reduce their success over the longer term. In the low-hunting regime, however, maternal parent trees were rarely found within 2 km of their offspring (<8% saplings; Fig. 2D). Without elephants, long-distance gene flow is lost, as the limited range of pollen dispersal cannot compensate for the drastically reduced seed dispersal. As ebony shifts from a spatial distribution that is indistinguishable from complete spatial randomness to a clumped pattern, it will encounter shifting mutualistic and predatory interaction dynamics at each stage of its life cycle, potentially affecting ecological and evolutionary processes (*48*).

In the Neotropics, some trees are thought to be adapted to megafaunal seed dispersal and to have survived the Pleistocene megafaunal extinction through various alternative dispersal mechanisms or surrogate dispersers (*34*, *47*). There is evidence that the distribution and abundance of these trees have been negatively affected by the change in the disperser community (*61*, *62*). Our results suggest that African ebony is transitioning from an elephant-dominated long-distance dispersal system to one where shorter-distance dispersal predominates. However, we found no evidence of hunting-resilient wildlife being substitute dispersers that could maintain ebony populations at levels similar to those supported by elephants. This is consistent with the identity of the primary agent of ebony seed removal in highly hunted forests observed during our study (figs. S6 and S7), Emin’s pouched rat (*C. emini*), which larder-hoards seeds within burrows that are too deep for successful germination (*63*).

Ebony is designated a vulnerable tree species under the International Union for Conservation of Nature (IUCN) Red List criteria due to its documented exploitation in Cameroon (*30*). We show that the extermination of forest elephants results in a severe additional threat to ebony that is large in magnitude and geographical scale. Our results suggest that regeneration, population, and gene flow declines are ongoing where the elephant is already absent, i.e., at least 65% of ebony’s historical distribution (Fig. 1A). These trends may worsen as the few remaining elephant populations collapse. The severe reduction in juvenile relative abundance under high-hunting regimes reflects a long-term recruitment bottleneck that now approaches the reproductive population (≥10 cm). In these hunted areas, adult survival becomes even more critical for maintaining the population, highlighting the importance of prioritizing adult tree protection in logging management strategies to ensure the species’ long-term viability.

We found no evidence that the mechanism underlying the ebony population decline is related to the escape from density-dependent mortality or improved germination after gut passage, as reported for other tree species. Instead, we found strong support for the benefit of pulp removal and a larger effect of a previously undocumented mechanism: Elephant dung physically protects ebony seeds from predators, directly supporting germination. The significant benefits of dung protection may be widespread among the numerous plant species dispersed by elephants. Their identification should, therefore, constitute a conservation priority as they form an essential part of the forest biomass (*9*, *64*) and are part of the cultural and economic heritage of the Congo Basin.

## MATERIALS AND METHODS

We collected samples and conducted research under permits issued by the Republic of Cameroon’s Ministry of Scientific Research and Innovation (MINRESI) and the Ministry of Forests and Wildlife (MINFOF). In addition, we obtained approval from the Conservator of the Dja Faunal Reserve (MINFOF), G. Oum Ndjock, who is also a co-author of this study. Research activities in the reserve were carried out under the supervision of the conservation service, with ecoguards actively participating in data and sample collection. The Dja Biosphere Reserve encompasses three of our research sites, including the Kompia site. Prior to fieldwork, our research plans and sample collection were discussed with the local community in Kompia, and access to the forest was granted by the Chief of Kompia. J. M. Ntsihe, from the Kompia community, and G. G. Mempong, from the Baka community of Bifolone, are co-authors of this publication in recognition of their expertise and active roles in shaping and implementing field protocols and managing logistical challenges during research activities. Access to the Mbalmayo site, located within the Mbalmayo Forest Reserve, was granted by the Director of the National Forestry School of Mbalmayo, Cameroon.

### Ebony and elephant range

The current known and possible ranges of forest elephants were derived from the IUCN database (*3*). The historical distribution of ebony was hand-digitized to include verified herbarium records (*29*). Large mangroves and swamp forests were derived from the ESA (European Space Agency) 2015 land cover map v. 2.0.7 (*65*), and areas above 1000 m elevation were derived from Shuttle Radar Topography Mission data. Both types of areas were excluded due to their unsuitability for ebony (*29*).

### Study sites

We selected four study sites of structurally intact forests along a gradient of increasing human pressure on fauna, hereafter referred to as sites A to D (detailed characteristics in table S1). Maintaining consistent habitat conditions helped avoid bias in parentage relationships and stem-diameter distributions. Ebony stumps from prior logging were extremely rare, with only two in site C and one in site D, despite the higher human density around those sites. Each stump was deemed recent (<10 years old), making them unlikely to influence our results.

Site A is located near the Bouamir Research Station in the Dja Faunal Reserve. Site B, near the Sim river, is inside the same reserve but closer to the edge. Poaching of ivory has reduced the elephant population in the reserve from 0.56 individuals km^−2^ in 1995 to 0.04 individuals km^−2^ in both 2018 and 2021 (*66*, *67*). Typical elephant densities in unhunted forests are usually estimated to be 0.5 to 1 individual km^−2^ (*13*, *68*, *69*). Site C, located within Kompia Community Forest, was chosen away from the main logging activities that began in 2006 (*70*). The village of Kompia had 296 inhabitants in 1967 and 687 in 2005 (*57*, *58*) and is located 0.7 km from the nearest plot corner. Essiengbot, with 244 inhabitants in 1967 and 671 in 2005, is 1.5 km away (*71*, *72*). Bushmeat hunting intensity is reduced in the reserve (A and B) compared to its exterior (C). Large mammals, including elephant, chimpanzee, and gorilla, went extinct in site C before 2001 and are still absent today (*56*, *73*). Hunting in site C is associated with double the camera trap detection rate of Emin’s giant pouched rat (*C. emini*) compared to sites A and B (*56*). Site D is in Mbalmayo Forest Reserve. The reserve has experienced substantial farming and silviculture since at least 1947 (*74*). We selected a remote area enclosed by the Nyong river where no recent human clearings have been observed. Mbalmayo City, located 10 km from the nearest plot corner, increased in population from 12,649 in 1965 to 52,809 in 2005 (*71*, *75*). Mammalian diversity is low, although a relict population of gorilla still persists (*76*). No evidence of elephant presence in sites C and D has been observed during fieldwork, either during the inventory or subsequent visits. Recent human population density was calculated using the 2005 national census data for villages and towns within a 20-km radius of the center of each forest inventory (*71*).

### Forest inventories and sampling

A comprehensive ebony tree inventory consisting of a square plot of 2 km by 2 km was established at each site. The edges of the plots were aligned with true north-south and east-west azimuth. A north-south transect was opened every 100 m. The forest within each pair of adjacent transects was inspected by six or more trained people to record every ebony tree discovered at least 130-cm tall. The dbh was measured at 130 cm above ground or above buttresses, if any. Smaller individuals were recorded to inform neighborhood models but were excluded from demographic analyses, as the inventory was not considered exhaustive for such small trees, which are often difficult to detect in the dense understory. In the rare case of multiple stems at measurement height, the largest one was selected. Geographical coordinates of every tree were obtained using Garmin GPSMAP 64s and 65s global navigation satellite system receivers. Azimuth and distance from a reference adult ebony tree were recorded instead of absolute coordinates when trees were less than 15 m apart. For parentage analyses, a leaf or piece of cambium was sampled and immediately dried in silica gel. To improve the accuracy of parameter estimates, embryos were opportunistically sampled from ebony seeds under seven mother trees in site C and processed as described above. Coordinates of transitions between terra firme forest, bare rocks, Raphia swamps, and farmlands along the transects were recorded during the inventories. Voucher specimens of *D. crassiflora* collected in each of the four sites were deposited at both the Cameroon National Herbarium and Université Libre de Bruxelles under accession numbers V. Deblauwe 315, 319, 377, 391, and 397.

To identify the sexual identity of ebony trees (pistillate or “female”; staminate or “male”; not flowering), we observed the flowering of each recorded tree of plots A, B, and C in March 2019 and 2020. Additional female trees were identified by recording fruit presence in August 2020 or abundant seedlings below the crown.

Diameter structures of ebony populations at each site were compared using a nonparametric two-tailed two-sample K-S test. Greenwood’s formula was used to compute 95% CI around the empirical cumulative distribution functions.

### Spatial point pattern

To characterize the spatial patterns of ebony trees, we calculated the univariate PCF, *g*(*r*), at the four study sites (*77*). The PCF represents the probability of observing a pair of points at a distance *r*, standardized by dividing by the probability expected under a Poisson process (i.e., random and independent spatial distribution of points). The function indicates whether the spatial distribution of points is clustered [*g*(*r*) > 1], overdispersed [*g*(*r*) < 1], or independent [*g*(*r*) = 1]. Because environmental variations within the study sites cause the tree distribution to deviate from uniform density (fig. S2), we estimated inhomogeneous PCFs with σ = 250 m (*78*). The inhomogeneous PCF standardizes the estimated density by the expected intensity of points given the inhomogeneous process. We tested the deviation from the null hypothesis of no association by comparing the observed values to the 5% two-tailed critical values from 999 Monte Carlo simulations of the null model. Observations below or above the simulated pointwise envelope were interpreted as trees being significantly overdispersed or clustered, respectively, at a given distance. We applied the PCF using the spatstat package v. 3.0-6 in R 4.3.0 with Ripley’s isotropic edge correction and a normalization power of two (*79*). The observation polygons included the square sampling plots, excluding environments where ebony trees were absent during exhaustive inventories (e.g., Raphia swamps and rocky outcrops), areas impacted by anthropogenic pressure (farmlands), and inaccessible areas (a portion of site D beyond the Nyong river). The effective sampled area is provided in table S1. We estimated two additional PCFs by pooling sites A and B together, and sites C and D together. In addition, each individual site was divided into two sections of equal area by drawing a straight division passing through the center of the plot. The pointwise average PCF, $\hat{g}\left(r\right)$ , and 95% CI were derived from these local functions using the varblock function.

### Microsatellite development

We developed microsatellites from a DNA extract of *D. crassiflora* following Micheneau *et al.* (*80*). This allowed us to select 17 loci containing perfect (i.e., not compound) microsatellites with at least nine dinucleotide (or trinucleotide for two loci) repeats and with primers located at least 20 base pairs from the microsatellite region. Fluorescent labeling was performed via amplification with the reverse primer, the forward primer with a Q1 to Q4 universal sequence at the 5′ end (“M13-tail”), and a Q1 to Q4 primer labeled with 6-FAM, NED, VIC, and PET, respectively (table S3) (*80*, *81*). We used the Type-it Microsatellite polymerase chain reaction (PCR) kit (QIAGEN) for multiplex amplification of two primer mixes of nine loci in a final reaction volume of 15 μl containing 7.5 μl of Type-it Multiplex PCR Master Mix, 3 μl of Q solution (5×), 0.1 μl of each forward primer (10 μM; but 0.2 μl for loci R11-002, R11-058, and R11-142), 0.15 μl of each reverse primer (10 μM; but 0.3 μl for loci R11-002, R11-058, and R11-142), 0.15 μl of labeled primers Q1 and Q3 (10 μM), 0.3 μl of labeled primers Q2 and Q4 (10 μM), and H_2_O. Multiplex PCR conditions were as follows: initial denaturation at 94°C (4 min); 24 cycles of denaturation at 94°C (30 s), annealing at 55°C (45 s), and elongation at 72°C (60 s); eight cycles of denaturation at 94°C (30 s), annealing at 53°C (45 s), and elongation at 72°C (60 s); and a final extension step at 60°C (30 min). For each PCR product, 1.2 μl was directly added to 12 μl of Hi-Di Formamide (Life Technologies, Carlsbad, CA, USA) and 0.3 μl of MapMarker 500 MW-0191-80ORANGE size standard (Eurogentec, Seraing, Belgium), then genotyped on an ABI 3730 sequencer (Applied Biosystems, Lennik, The Netherlands). The number of alleles per locus and observed and expected heterozygosities were calculated using SPAGeDi v. 1.5d (*82*). The genotype of two adult trees in site C, one dead for several decades and one logged, were reconstructed based on inferences from progeny found within 10 m of the stump.

### Fine-scale spatial genetic structure

The FSGS of the ebony populations within and around the sampled plots (sites A, B, and C) was characterized by the decay of the kinship coefficient *F* with spatial distance *r* (kinship-distance curve), following Vekemans and Hardy (*44*). Given the lower abundance in sites A and B, we pooled both sites for analysis. We used the software SPAGeDi v. 1.5d to estimate the J. Nason’s estimator (*83*) *F_ij_* for pairs of individuals *i* and *j* in forests with low hunting (pooled sites A and B) and high hunting pressure (site C), separately for small saplings, large saplings, and adults. We used nonoverlapping bins of pairwise spatial distances to average the estimates of *F_ij_* and visualize the *F*(*r*) curve (bin edges of 0, 30, 100, 200, 400, 800, 1600, and 3200 m). Standard errors were calculated by jackknifing loci.

The set of *F_ij_* values were regressed on the spatial distance ln(*r_ij_*) between individuals providing the regression slope *b_Ld_*. The latter can inform about the amplitude of FSGS through the statistic *Sp*, which synthesizes the decay of kinship coefficient between individuals with distance (*44*). The significance of *F*(*r*) and *Sp* was tested by comparing the observed values with 95% envelopes computed from 4999 Monte Carlo permutations of the spatial positions of the trees (no genetic structure). We compared the means of *b_Ld_* values of loci (*n* = 18 pairs of loci) using two-sided paired-sample *t* test as a proxy to compare *Sp* statistics.

### Seed and pollen dispersal parameters

We relied on the neighborhood model implemented in the software NMπ v. 2.0 (*84*) to model the forward seed and pollen dispersal kernels by a maximum-likelihood approach accounting simultaneously for genotypic and spatial data of adults and juveniles. The sex of trees when known from the observation of flowers or fruits was included as input. The genotypes of the seeds from known mothers in sites A and C were included to improve the accuracy of genotyping error rates and pollen dispersal kernel estimates. Samples with fewer than six amplified loci were not included in the analysis. We fitted the following pollen and seed dispersal parameters: (i) the selfing rate, *s*_o_; (ii) the pollen and seed immigration rates, *m*_p_ and *m*_s_, indicating the proportions of seeds and saplings sired or mothered by nonsampled adults (i.e., parents situated outside of the sampled areas or inside the sampled areas but missed during the sampling); and (iii) two parameters of the exponential power–von Misses distribution for modeling the pollen and seed dispersal kernels. These kernel parameters were (i) the reciprocal of the mean pollen and seed dispersal distances, *d*_p_ and *d*_s_, and (ii) the exponents, *b*_p_ and *b*_s_, controlling the shape of the distributions (i.e., *b* = 2 for a Gaussian distribution, *b* = 1 for an exponential distribution, and *b* < 1 for a fat-tailed distribution). From the occasional parent-progeny genotypic mismatches occurring in the data, NMπ calculated genotyping error rates per locus. Because of the smaller number of samples in plots A and B, genotyping error rates calculated in plot C were used instead of their own. From the parameters, NMπ assigned the most probable mother and father to each seed and sapling, with a probability value. Considering cases where mothers were inferred with a probability of ≥0.5, we compared the distributions of seed dispersal distances and immigration rates between low- and high-hunting pressure sites.

### Density- and distance-dependent mortality of seeds and seedlings

Seed and seedling distributions were manipulated in forests with low and high hunting pressure. Visually intact seeds were collected from ripe fruits under adult trees at the peak of fresh fruit abundance, washed with water, stored for less than 48 hours in jute bags disinfected with boiling water, and manually dispersed at set distances from the nearest adult trees at sites A and C. To control for the distance from sampled trees to the nearest mature tree, adult trees were subsampled from those identified during the exhaustive inventory of the plot. To calculate the distance to the nearest adult, we selected trees with a dbh of ≥20 cm that were spaced at least 100 m from both the nearest conspecific adult (dbh of ≥20 cm) and plot edges. The remaining 14 and 25 trees in sites A and C, respectively, were used as sampling units. From each tree, we established two virtual transects pointed in opposite directions. We placed seeds in 1-m^2^ square subplots along each transect at distances of 2, 20, and 50 m from the center of the mature tree (Fig. 4C). Although elephants disperse most seeds over distances exceeding 1 km, we limited our study to shorter distances because the influence of conspecific plants in tropical forests generally extends only up to 15 m (*57*). Each subplot contained two seeds for one transect and 25 seeds (20-cm spacing in a 5-by-5 square grid) for the other transect. The fate of each seed (*n* = 3159 seeds) was recorded 2 months after the experimental setup as one of the following categories: intact, removed, physically damaged (chew marks or insect holes), rotten (fungi developed on the seed), germinated (radicle has extended outside the seed shell, but the two leaflets have not fully deployed), or established (the two leaflets are fully deployed outside of the seed shell). To identify mammalian seed predators, we placed camera traps in front of the 25-seed subplot from the 2-m treatment under 10 and 14 trees in sites A and C, respectively.

Seeds from the same sources were protected from predators and germinated in temporary nurseries. One month later (end of November to early December), the germinated seeds were planted around 6 and 14 of the same adult trees in sites A and C, respectively, as in the seed experiment, but in transects orthogonal to the seed transects. On each transect, we placed seedlings in subplots located at distances of 2, 20, and 50 m from the center of the mature tree. On one transect, the seedling plot contained two seedlings per 1-m^2^ subplot, and on the other, 25 seedlings per 1-m^2^ subplot (20-cm spacing in a 5-by-5 square grid). The fate of each seedling was recorded 1 year after the experimental setup as one of the following categories: intact, removed, physically damaged (chew marks or insect holes), or dead. To identify mammalian herbivores, we placed camera traps in front of the 25-seedling subplot from the 2-m treatment under the 6 and 14 adult trees in sites A and C, respectively.

### Seed environment

The effect of the primary dispersal mode on secondary seed fate was investigated by manipulating in situ seed environments. Visually intact seeds were collected from ripe fruits under adult trees at the peak of fresh fruit abundance and deposited in the forest in three groups of five seeds mimicking undispersed, rodent-dispersed, and elephant-dispersed seeds, respectively: (i) seeds contained in fruit, (ii) naked seeds on the ground, and (iii) seeds in elephant dung (Fig. 4B). Seeds in (ii) and (iii) were washed with water and stored for less than 48 hours in jute bags disinfected with boiling water. Germination rate after different primary dispersal modes was investigated by doubling each of the three treatments in a mammal exclusion cage, 120 cm by 40 cm by 30 cm, made of 2.5 cm–by–1.5 cm steel mesh. To investigate the effect of gut passage on germination, a seventh treatment was created in the exclusion cage with seeds found in dung placed in an artificial dung pile (Fig. 4B). The fruit treatments involved using a whole fruit from which any seed exceeding five were removed by making a transverse incision in the upper half of the exocarp, which was then replaced before placing the fruit on the ground. The dung treatment consisted of dung material collected from the nearby forest and stored in the shade for less than 48 hours. Each group was separated by ≥6 m, and the three treatments were replicated 60 times at an interval of ≥100 m at ≥10 m from a forest track (*n* = 250 seeds in each treatment). Sites unsuitable for ebony survival (swamps, logs, and forest openings) were discarded as possible experimental locations. Extremely dense thickets were also discarded as elephants open the forest as they walk. Thus, our results should be considered indicative of realistic elephant dispersal but cannot rule out an interaction effect of shading, competition, or microenvironment conditions.

The fate of each seed was recorded 2 months after the experimental setup as one of the following categories: removed, germinated (the two leaflets are fully deployed outside of the seed shell), or not germinated (none of the above). To identify mammalian seed predators, camera traps were placed in front of each treatment in eight replicates, ≥200 m apart (*n* = 120 seeds).

### Generalized linear mixed-effects model

To quantify and assess the effects of treatments on the binomial responses of seeds (removed/germinated or not) and seedling fates (survival or not), we fit generalized linear mixed models with a logit link and the Laplace approximation technique of parameter estimation using the lme4 v. 1.1.34 package in R. When modeling density-and distance-dependent mortality of seeds and seedlings, our global models included the fixed effects of density categories plus the interaction between distance to the nearest adult (continuous in meters, hereafter “distance”) and hunting pressure (low versus high). The global model for the in-cage dung experiment included treatment categories (fruit, dung, ground, and dung+elephant gut) only; for the out-of-cage dung experiment, the global model included treatment and year (2021 or 2022).

To control for potential spatial dependence between replicated treatments during the experiments, we included a random effect of sampling unit. To control for dependence at a broader spatial scale, if any, we nested the effect of sampling units (adult trees) within the random effect of watershed. For the model of density and distance dependence, watershed (*n* = 4 in A and *n* = 6 in C) was nested within each of the two hunting pressure regimes. Models did not converge for the seed density- and distance-dependence experiment when we included the random effect of watershed, so we simply used a sampling unit random effect. We ran a post hoc model identical to the best-fit model for seedlings but excluding nested watershed effects. An LRT detected no difference between this simpler model and the best-fit model with the nested random effects (LRT: *P* = 0.85, χ*^2^* = 0.017, df = 1). Because the same adult trees were used in the seed and seedling experiments, we have no evidence to suspect that the lack of a nested random effect had any effect on seed fate.

For each of the four mixed-model analyses, we performed model selection using LRTs at α = 0.05. We started with a global model and proceeded stepwise, comparing the more complex model with a simpler nested model that excluded the least supported fixed effect. We based our conclusions on model selection as well as 95% CI estimates generated and graphed from the best-fit models. Goodness-of-fit for best-fit models is reported as the variance explained by the fixed effects, or marginal *r^2^* for generalized mixed models, using the delta method in the R package MuMIn v. 1.47.5 (*85*). We report generalized linear mixed model *t* statistics and *P* values calculated from *z* distributions and provide results from Tukey’s tests (R package emmeans v. 1.8.8) for pairwise comparisons between treatment levels. For the seedling survival analysis, we included hunting and density even though their coefficients were marginally significant (0.05 < *P* < 0.065) because they were central to our ecological questions and because the full additive model (distance + hunting + density) fit significantly better than the univariate model with distance alone (LRT: *P* = 0.028, χ*^2^* = 7.146, df = 2; additive: *r^2^* = 0.050; univariate distance: *r^2^* = 0.048).

## Supplementary Materials

### This PDF file includes:

Figs. S1 to S8 Tables S1 to S6 References
