# Supplementary material for: Declines of ebony and ivory are inextricably linked in an African rainforest
Source: Sci Adv. 2025 Aug 27;11(35):eady4392. doi: 10.1126/sciadv.ady4392 (PMC12383251; doi:10.1126/sciadv.ady4392)
Supplement: Supplementary file 1 — Figs. S1 to S8 Tables S1 to S6 References [file sciadv.ady4392_sm.pdf]

Science Advances
AAAS

Supplementary Materials for

Declines of ebony and ivory are inextricably linked in an African rainforest

Vincent Deblauwe *et al.*

Corresponding author: Vincent Deblauwe, v.deblauwe@cgiar.org

Sci. Adv. **11**, eady4392 (2025)
DOI: 10.1126/sciadv.ady4392

This PDF file includes:

Figs. S1 to S8
Tables S1 to S6
References

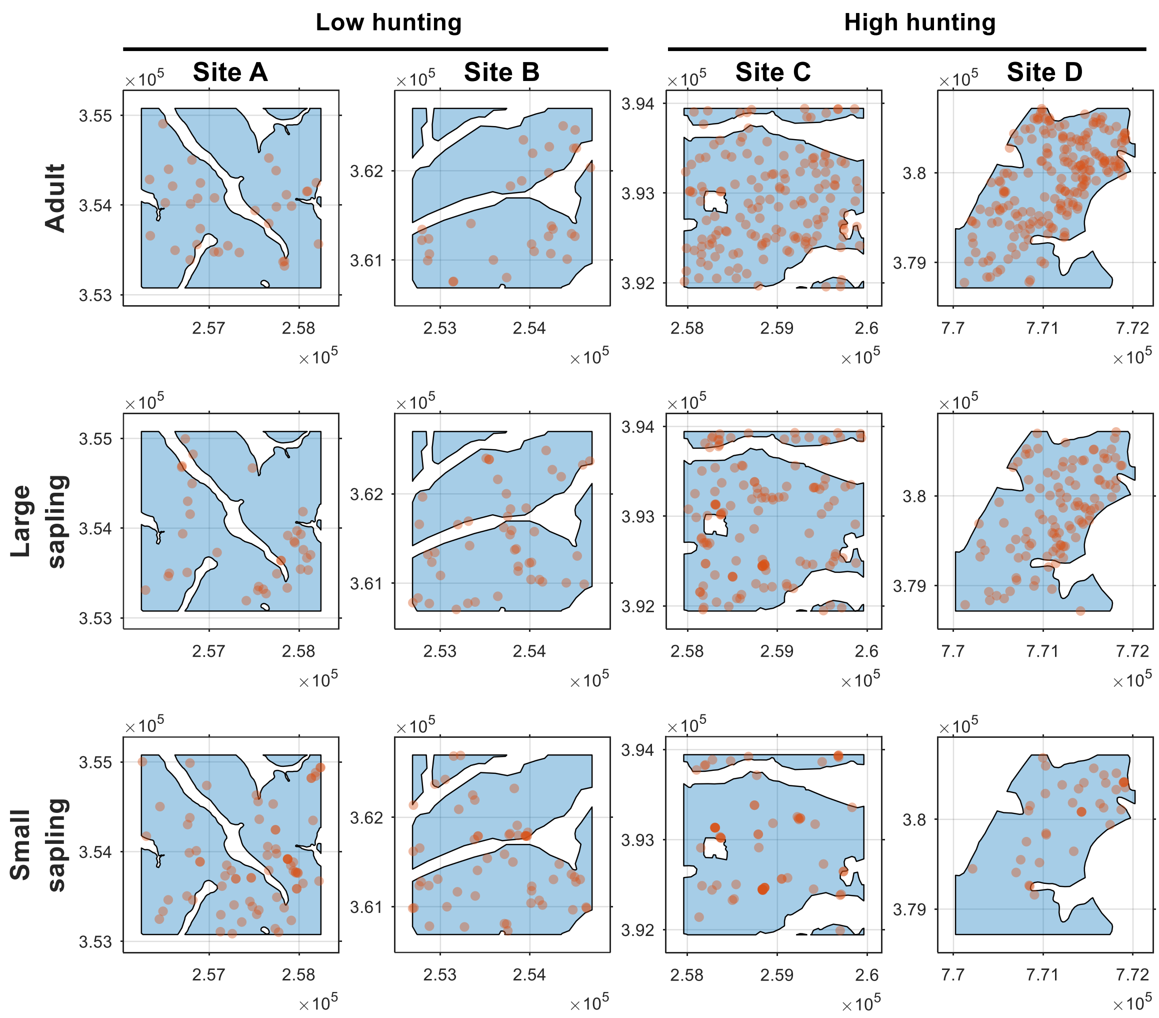

Fig. S1. Maps of *Diospyros crassiflora* individuals by cohort across the four sites. Cohorts of non-overlapping dbh classes (top to bottom, a, 10 cm ≤DBH; b, 5 cm ≤DBH≤10 cm; c, 2 cm ≤dbh≤5 cm) of ebony trees (red) from four populations (left to right, sites A, B, C, D) are represented with the forest suitable for ebony (blue). Coordinates are given in meters, UTM projection, datum WGS84.

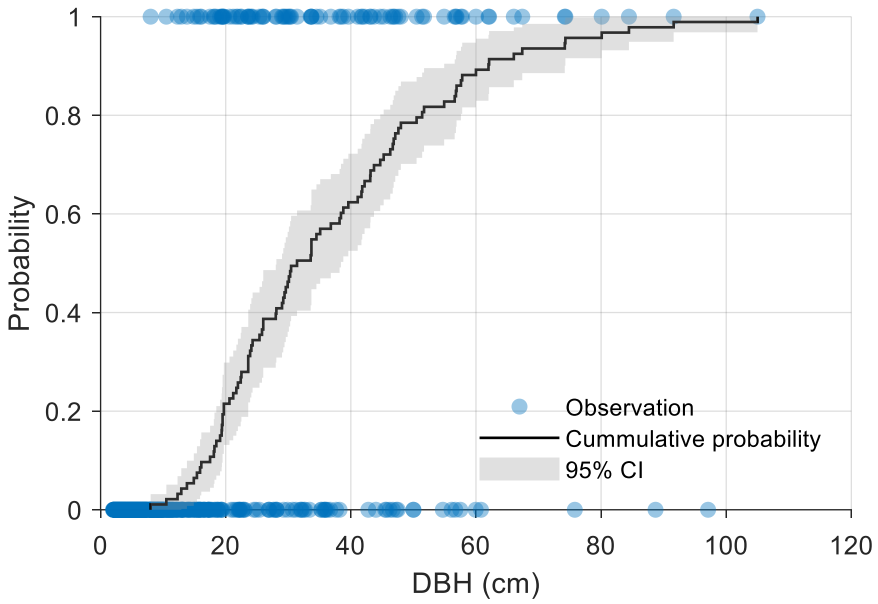

Fig. S2. Cumulative probability of flowering in relation to *Diospyros crassiflora* stem diameter in plot C. The probability of trees being observed producing flowers (pistillate or staminate) at least once over two years is shown as a function of main stem diameter. Empirical cumulative distribution functions among flowering trees (solid black) and 95% confidence intervals estimated by using Greenwood's Formula (grey area) are shown. Individual tree flowering states as blue dots.

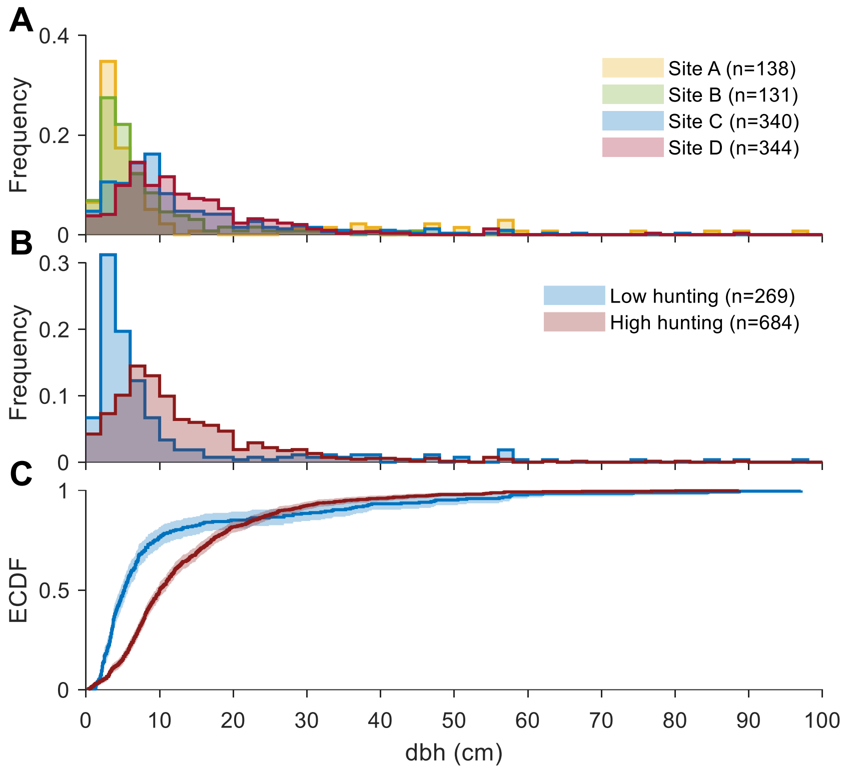

Fig. S3. *Diospyros crassiflora* population dbh structure in low and high hunting regimes. Population dbh structure of ebony in the four study sites (**A**) and merged into protected versus defaunated forests (**B**) showing the 'missing cohort' of small ebony saplings coinciding with the period when vertebrate dispersers were lost. (**C**) Empirical cumulative distribution functions (ECDF) of both populations (line) with 95% confidence intervals (area).

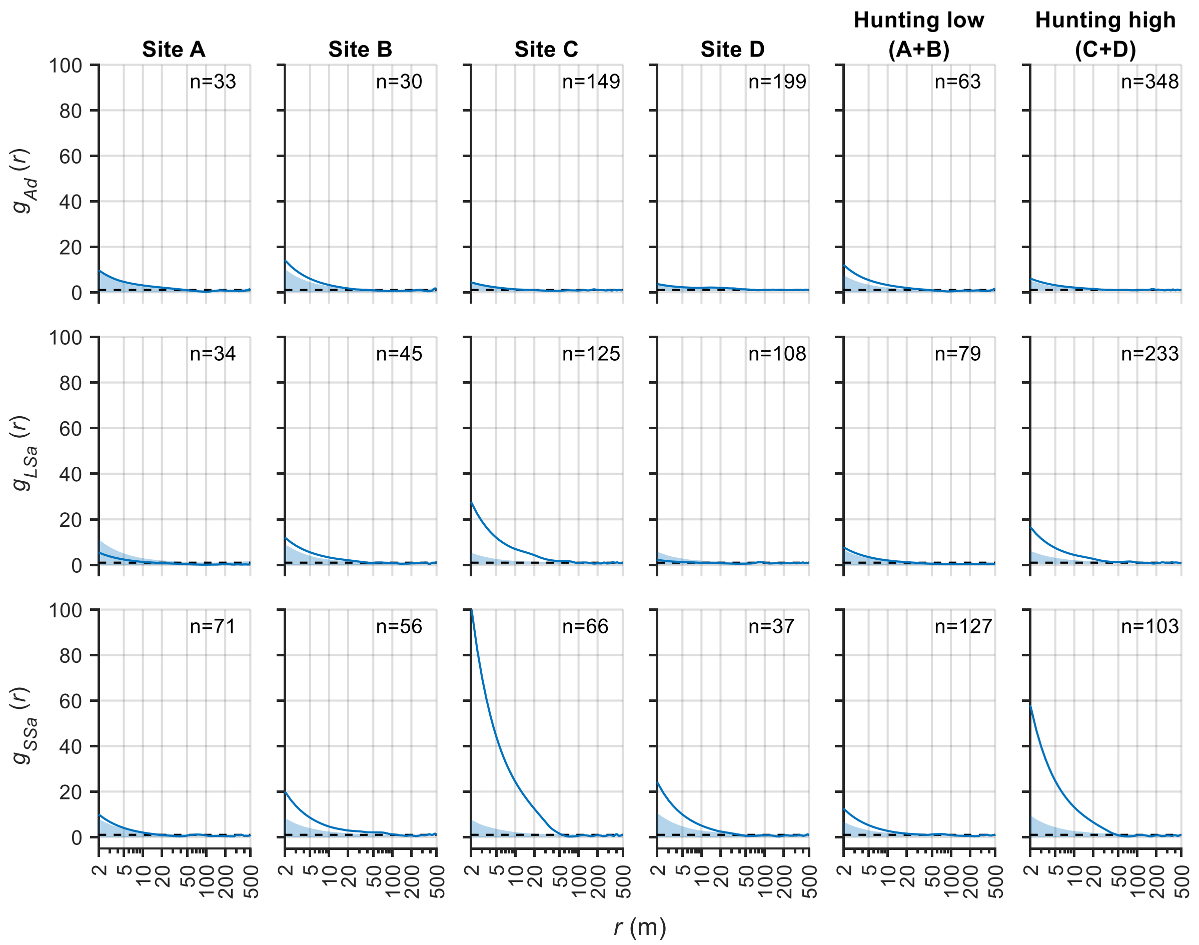

Fig. S4. Spatial clustering of *Diospyros crassiflora* by cohort, site and hunting pressure. Inhomogeneous univariate pair correlation function (PCF, blue line), *g* (*r*), is the observed number of pairs of points in the pattern that are about r apart, divided by the expected number that would be obtained if the points were completely random. Top to bottom, adults (Ad, dbh ≥ 10 cm); large sapling (LSa, 5 cm ≤ dbh <10 cm) and small sapling (SSa, 0 cm ≤ dbh <5 cm) in four populations (A to D), pooled low-hunting (A, B) and pooled high-hunting (C, D) forests. PCFs should be compared across cohorts within the same site to control for the influence of adult density. In this context, both hunted sites (C, D) exhibit increasing levels of clustering from small to large tree diameters. In contrast, both protected sites (A, B) show relatively constant levels of clustering across cohorts. The 95% envelope (blue area) of the null hypothesis of lack of association ($g = 1$, dashed line, Poisson process) was calculated from Monte Carlo simulations.

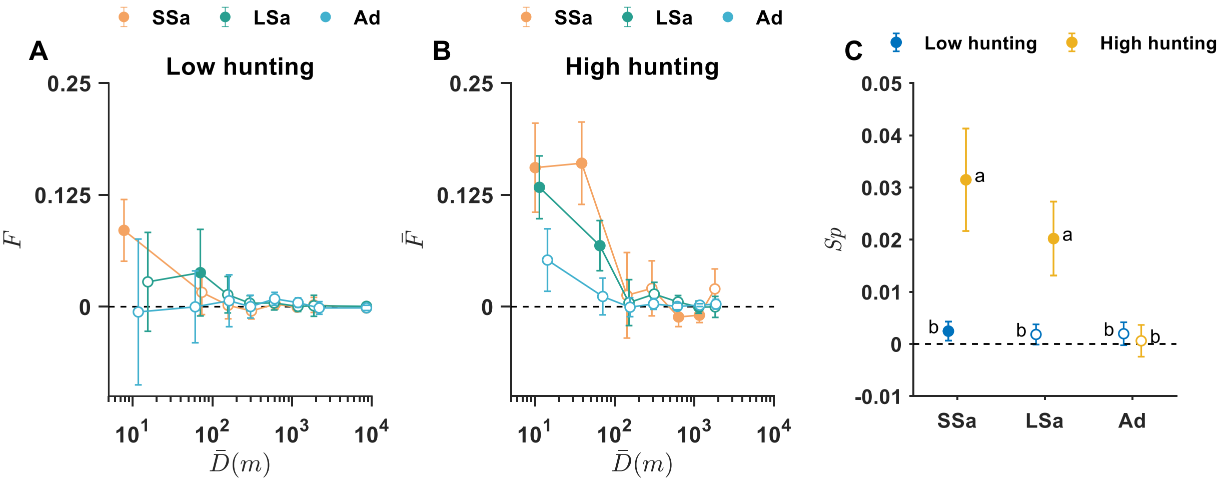

Fig. S5. Fine scale spatial genetic structure (FSGS) of *Diospyros crassiflora* by cohort and hunting regime. Average kinship coefficient, $F(r)$, as a function of distance, D (log scale), within three cohorts: adults (Ad), large saplings (LSa) and small saplings (SSa) averaged within distance bins. (**A**) Low-hunting forest (sites A and B) showing significant kinship among small saplings at short distance. The largest distance class represents coefficients between individuals in sites A and B. (**B**) High-hunting forest (site C) showing significant kinship within both small and large saplings at short distance, which can be compared to the expected F_{ij} of half-siblings (1/8) and full siblings (1/4). (**C**) Magnitude of FSGS of each cohort expressed by the *Sp* statistic calculated from the rate of decrease of pairwise kinship coefficients between individuals as a function of ln(D). Letters for statistically significant differences (two-tailed paired-sample *t*-test with Holm-Bonferroni correction, n=18 loci, $P \leq 0.05$). Whiskers for 95% CI estimated as the normal approximation interval based on the standard error estimated by jackknifing loci. Pointwise F_{ij} and S_p difference ($\alpha = 0.05$) with expected value (dashed line) under absence of FSGS (spatial permutation test) are shown as closed circles. Open circles indicate non-significant difference. SSa, small sapling; LSa, large sapling.

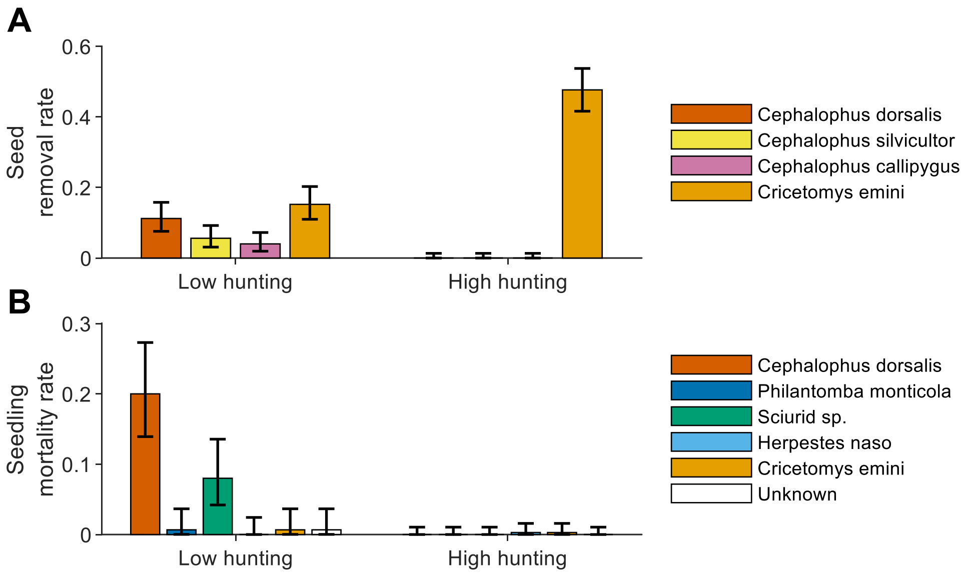

Fig. S6. Seed and seedling removal and mortality by agent under contrasting hunting pressures. Fresh seeds and one-month-old seedlings fate observed by camera traps for one month after deployment. (**A**) The number of seeds removed and predated was normalized by the total number of seeds observed (n=250 and 275 in low and high hunting treatments, respectively). (**B**), Number of seedlings killed during first month after transplantation, normalized by the total number of seedlings observed (n=150 and 350 in low and high hunting treatments, respectively). Events occurring after the camera trap ended recording were characterized as “unknown”. Error bars indicate 95% confidence intervals calculated with Clopper-Pearson method.

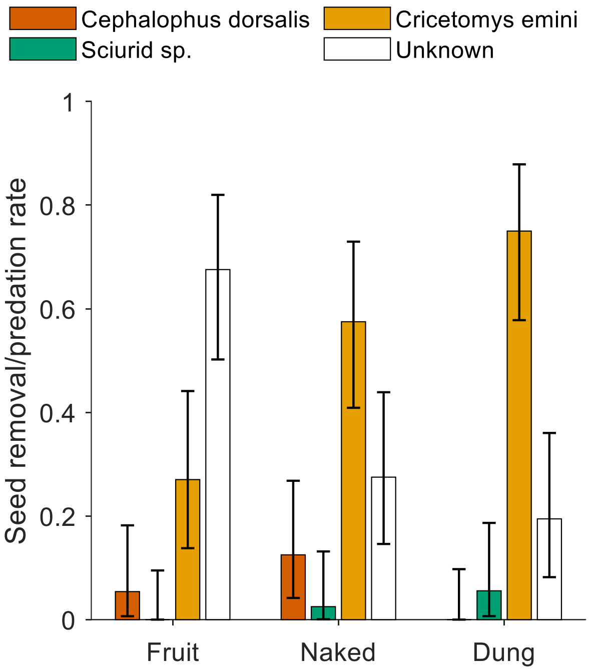

Fig. S7. Seed removal/predation by agent across primary dispersal treatments. Data were normalized by the number of seeds removed or predated. The treatments mimic undispersed seeds (fruit pulp), seeds dispersed by ungulates or rodents (ground), and seeds dispersed by elephants (dung pile). The fate of 40 seeds per treatment was observed for one month. Seeds removed after the camera trap ended recording were characterized as "unknown". Error bars indicate 95% confidence intervals calculated with Clopper-Pearson method.

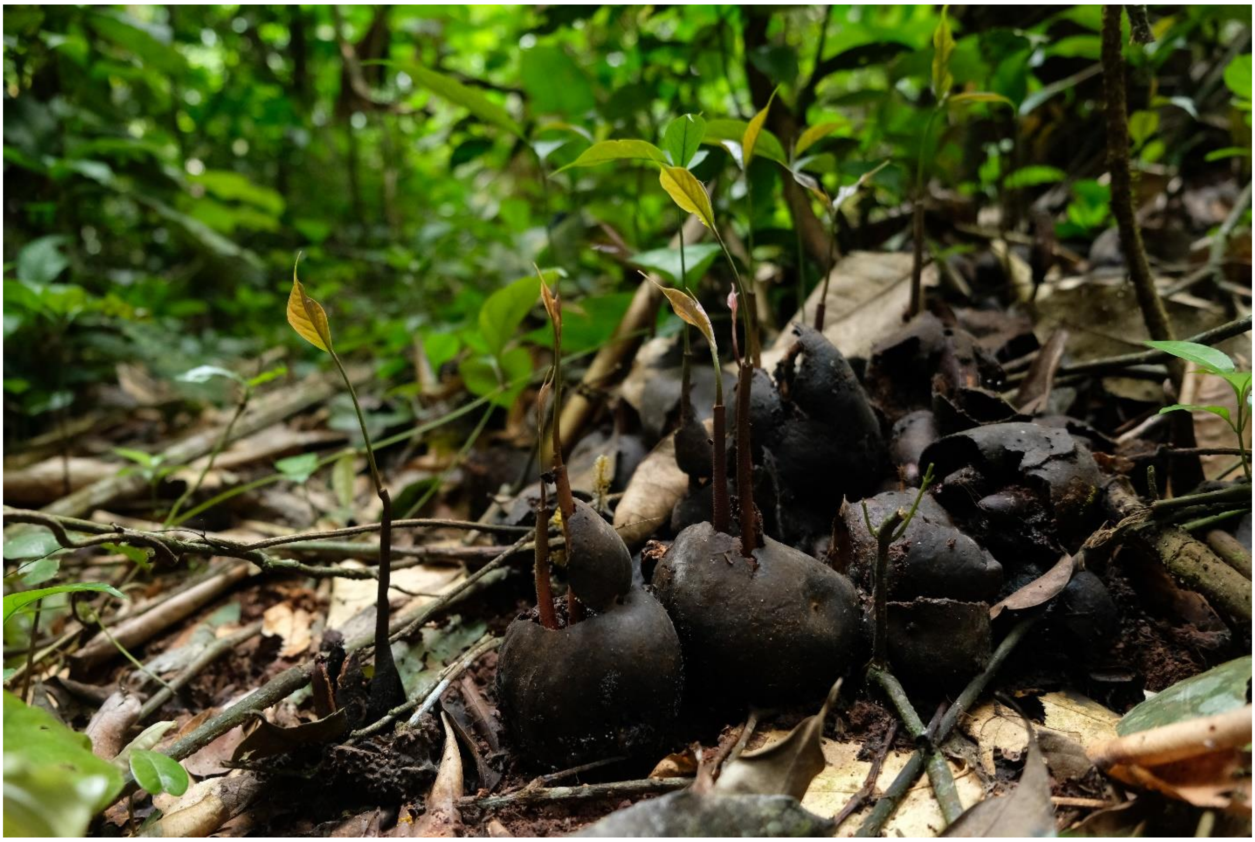

Figure S8. Ebony seeds germinating in decomposing fruits beneath the mother tree in Site C. Photo credit V. Deblauwe.

Table S1. Description of the study sites.

Plot Name	Plot A	Plot B	Plot C	Plot D
Nearest landmark	Bouamir research station	Petit rocher, Sim river	Kompia	Mbalmayo
Latitude (N)	3°11′31.2″	3°15′39.2″	3°32′36.3″	3°25′23.6″
Longitude (E)	12°48′23.7″	12°46′28.3″	12°49′16.4″	11°25′48.8″
Effective surface (ha)	349.2	326.1	308.4	258.4
Relative hunting pressure	Low (*56*)	Low (*56*)	High (*56*)	High (near major city)
Elephant density (ind. km^{-2})	0.04 (2018) (*66*)	0.04 (2018) (*66*)	0	0
Forest type	humid evergreen	humid evergreen	humid semideciduous with some evergreen forests	humid evergreen with some semi-deciduous forests
Land classification	Faunal reserve	Faunal reserve	Community forest	Forest reserve
Elevation (m)	675–720	655–700	670–775	635–675
Mean annual temperature (°C)	23.2	23.5	23.1	23.2
Mean annual rainfall (mm)	1650	1565	1615	1540

Latitude and Longitude are given for the SW corner of the square plot inventory. Forest types from Letouzey’s classification of the Guineo-Congolean domain (*88*). Climate variables obtained by the Chelsa dataset v 2.1 (averaged from 1981 to 2010) (*89*).

Table S2. Overview of data sources and methods used to assess ecological processes.

Ecological process	Method used	Sample size	Study site A	B	C	D
Regeneration/demography	Diameter/age distribution	138 (A), 131 (B), 340 (C), 344 (D) trees	✓	✓	✓	✓
Flowering	Census of all trees > 10 cm in dbh	93 flowering trees	✓	✓	✓	
Primary dispersal	Camera traps	11 trees (C), 9 trees (A), 16 fruits per camera	✓		✓	
Seed dispersal	Pair correlation functions	71 SSa, 34 LSa, 33 Ad (A); 56 SSa, 45 LSa, 30 Ad (B); 66 SSa, 125 LSa, 149 Ad (C); 37 SSa, 108 LSa, 199 Ad (D) trees	✓	✓	✓	✓
Seed and pollen dispersal	Neighborhood model (NMπ) and fine-scale spatial genetic structure (SPAGeDi)	304 (A+B), 469 (C) trees	✓	✓	✓	
Seed predation	3 Experimental treatments (fruit, ground, dung)	5 seeds x 3 treatment x 50 seed groups	✓			
	Camera traps during above experiment	5 seeds x 3 treatments x 8 seed groups				
	3 Experimental treatments (fruit, ground, dung)	5 seeds x 3 treatment x 60 seed groups	✓			
Germination	4 Experimental treatments in exclusion cages (fruit, ground, dung, dung+elephant gut)	5 seeds x 3 treatment (fruit, ground, dung) x 60 seed groups; 44 seeds in 9 groups (dung+elephant gut)	✓			
Distance and density dependent mortality of seeds	Experimental treatments (2 distances, 2 densities)	2 distances x 2 densities x 14 trees (C), 25 trees (A)	✓		✓	
	Camera traps during above experiment	10 (C), 14 (A) trees	✓		✓	
Distance and density dependent mortality of seedlings	Experimental treatments (2 distances, 2 densities)	2 distances x 2 densities x 6 trees (C), 14 trees (A)	✓		✓	
	Camera traps during above experiment	6 (C), 14 (A) trees	✓		✓	

SSa, small sapling; LSa, large sapling.

Table S3. Primer sequences for 17 microsatellite loci used to genotype *Diospyros crassiflora*. Q1 to Q4 are the sequences of the four possible tails added to the 5' end of the forward primers to integrate a fluorescent dye (respectively, FAM, NED, VIC, NED) to the PCR products. Multiplexed PCR was performed in two mix. The GenBank accession numbers, range of allele sizes, total number of alleles (*Na*), and expected (*He*) and observed (*Ho*) heterozygosity are given.

Locus	Primer sequence (5`-3`)*	GenBank	Labelled primer	Allele size (bp)	*Na*	*He* *Ho*
R11-002‡	F: Q1-AGGGCAGAAAGTGGTGAGAA	PV620819	Q1-FAM	135–175	9	0.74
	R: CCAATCTATTTGGAGGCATTC					0.73
R11-003†	F: Q1-TACCTCTTCTCCCATGTGCC	PV620820	Q1-FAM	142–164	11	0.76
	R: TGACCTTTCTGTCGTGAGTTG					0.69
R11-027‡	F: Q1-AAATTAAAATGCCCATCATAGCC	PV620821	Q1-FAM	245–272	12	0.78
	R: TGTAGTGCTTCTCTATTCTCAGCC					0.77
R11-031†	F: Q1-ATGGAGGCGGCTAAAGCTA	PV620822	Q1-FAM	291–320	4	0.47
	R: GACTGACAACTCAGATGCGG					0.46
R11-036†	F: Q1-CATGATGAGATGATGAGCCG	PV620823	Q1-FAM	204–225	6	0.70
	R: CCGAATGGGTTACAAGGAGA					0.71
R11-040†	F: Q2-CAGCCATGTGGATGGAGATT	PV620824	Q2-NED	137–170	11	0.68
	R: TTGGACAATTTGGCCTTTTG					0.63
R11-046‡	F: Q2-CCTCAGTTTTGTATGGTTACCG	PV620825	Q2-NED	163–169	4	0.21
	R: GTTGTGATACGAGCCTCCTC					0.14
R11-053‡	F: Q2-GCGACCAACTTCATCAAACC	PV620826	Q2-NED	185–204	8	0.63
	R: CCACCCACCTGTGGATTTAG					0.29
R11-058†	F: Q2-AGAAAATAGTGTTGTTATCGATCTAGG	PV620827	Q2-NED	210–232	10	0.83
	R: TGAGATTTCAATTGTGTTATTCCA					0.57
R11-065‡	F: Q2-TTCTAAAGCTTTTCTTCAAAGTCTTG	PV620828	Q2-NED	251–288	11	0.81
	R: AGCATTGTGCAAGGGGATAA					0.74
R11-070†‡	F: Q2-ACGCGTTCCAACTGGAAATA	PV620829	Q2-NED	317–338	7	0.78
	R: GGAAGCAACTGGAATGGAAA					0.71
R11-089†	F: Q3-GAGGCTGAAGAGGGAGAACA	PV620830	Q3-VIC	179–187	5	0.54
	R: TGCTGTAATCGACTGTACCTTTTC					0.54
R11-108‡	F: Q3-TGTGGCACATTTACAGTGAATCAA	PV620831	Q3-VIC	185–210	6	0.64
	R: TGCGGTTATGTTTGTTTCTTTT					0.66
R11-120‡	F: Q4-TTGGGGAAGAATGAGGACA	PV620832	Q4-PET	163–185	11	0.84
	R: GGGGAAGGGGTTCATCTAAA					0.80
R11-125†	F: Q4-CTGCCAGGAAGCTTGTGTTT	PV620833	Q4-PET	192–215	9	0.77
	R: CCACGAACATTGCCCTAAAT					0.72
R11-129‡	F: Q4-AAGGTTTTCGTGACTTTTCCA	PV620834	Q4-PET	212–227	6	0.69
	R: CCCTTATTTGGGACCACCTC					0.62
R11-142†	F: Q4-AAAGCAATTAAGAAAAGCTATGAATGA	PV620835	Q4-PET	296–334	13	0.68
	R: TTCGAAGCCTATCAAGAGCA					0.65

*Q1 = TGTAAAACGACGGCCAGT (*81*); Q2 = TAGGAGTGCAGCAAGCAT; Q3 = CACTGCTTAGAGCGATGC; Q4 = CTAGTTATTGCTCAGCGG (*90*). † multiplex 1; ‡ multiplex 2.

Table S4. Seed and pollen dispersal parameters of *D. crassiflora*. The neighborhood model was implemented in NMπ.

	**Low hunting (sites A+B)**	**High hunting (site C)**
**Sample size**		
n_{Adult}	76 (6)	154 (6)
$n_{Large\ sapling}$	81 (7)	97 (7)
$n_{Small\ sapling}$	123 (4)	70 (4)
$n_{Seedlings}$	23 (1)	148 (5)
n_{Seeds}	39 (0)	89 (0)
**Maximum likelihood estimate [95% CI]**[a]		
s_o	0	0
m_p	0.211 [0.088, 0.335]	0.407 [0.348, 0.465]
m_s	0.840 [0.786, 0.895]	0.164 [0.116, 0.211]
d_p	0.00221 [0.00156, 0.00286]	0.00388 [0.00337, 0.00439]
d_s	0.00048 [0, 0.00124]	0.00081 [0.00050, 0.00112]
b_p	1	1
b_s	0.2	0.2
γ	0.208 [0.052, 0.363][b]	0.208 [0.052, 0.363]
β	0.830 [0.695, 0.965][b]	0.830 [0.695, 0.965]

Total sample sizes are indicated, with the number of individuals from outside the plot in brackets. s_o, selfing rate; m_p and m_s, respectively, pollen and seed immigration rates; d_p and d_s, respectively, reciprocal of the mean pollen and seed dispersal distances; b_p and b_s, respectively, exponents controlling the shape of the pollen and seed distributions; γ and β, effect of dbh on female and male fitness, respectively. To achieve convergence, b_p and b_s were fixed to 1 and selection gradients (γ, β) were fixed to 0 in sites A+B. Number of samples in each cohorts that amplified are indicated.

[a]The confidence interval estimated as the normal approximation interval based on the standard error.

[b]Taken from site C due to lack of convergence.

Table S5. GLMM model selection results for germination in exclusion cages and seed survival outside cages.

Response	Year	Treatment	$\chi2$	df	*P*	Nbr parameters
Germination		✓				6
			**371.35**	**3**	**$3.54x10^{-80}$**	**3**
Seed survival	✓	✓				6
		**✓**	**0.66**	**1**	**0.416**	**5**
		✓				5
			259.45	2	4.57E-57	3

All models included the random effect of seed group and nested random effect of seed group within watershed. Best fit models in bold.

Table S6. GLMM model selection results for the density- and distance-dependent mortality of seeds and seedlings.

Response	Density	Distance	Hunting	Distance *hunting	$\chi 2$	df	*P*	Nbr params
**Seed survival**	✓	✓	✓	✓				6
		**✓**	**✓**	**✓**	**0.01**	**1**	**0.931**	**5**
		✓	✓	✓				5
		✓			10.14	2	0.006	3
		✓	✓	✓				5
		✓	✓		6.55	1	0.010	4
		✓	✓	✓				5
					18.80	3	$3.01x10^{-04}$	2
**Seedling survival**	✓	✓	✓	✓				
	✓	✓	✓		0.04	1	0.848	7
	✓	✓	✓					6
		✓	✓		3.50	1	0.061	6
		✓	✓					5
		**✓**			**3.64**	**1**	**0.056**	**4**
		✓	✓					5
					25.44	2	$2.99x10^{-06}$	3

Models for seed survival included the random effect of adult tree. Models for seedling survival included the random effect of adult tree and a nested random effect of adult tree within watershed. Best-fit models (repeatedly shown to demonstrate stepwise model selection) are in bold.

REFERENCES AND NOTES

1. R. T. Corlett, The shifted baseline: Prehistoric defaunation in the tropics and its consequences for biodiversity conservation. *Biol. Conserv.* **163**, 13–21 (2013).

2. E. C. Fricke, A. Ordonez, H. S. Rogers, J.-C. Svenning, The effects of defaunation on plants' capacity to track climate change. *Science* **375**, 210–214 (2022).

3. K. S. Gobush, C. T. T. Edwards, F. Maisels, G. Wittemyer, D. Balfour, R. D. Taylor, "*Loxodonta cyclotis* (errata version published in 2021). The IUCN Red List of Threatened Species 2021" (e.T181007989A204404464, IUCN, 2021).

4. J. R. Poulsen, C. Rosin, A. Meier, E. Mills, C. L. Nuñez, S. E. Koerner, E. Blanchard, J. Callejas, S. Moore, M. Sowers, Ecological consequences of forest elephant declines for Afrotropical forests. *Conserv. Biol.* **32**, 559–567 (2018).

5. C. Rosin, K. K. Beals, M. W. Belovitch, R. E. Harrison, M. Pendred, M. K. Sullivan, N. Yao, J. R. Poulsen, Assessing the effects of elephant foraging on the structure and diversity of an Afrotropical forest. *Biotropica* **52**, 502–508 (2020).

6. S. Blake, S. L. Deem, E. Mossimbo, F. Maisels, P. Walsh, Forest elephants: Tree planters of the Congo. *Biotropica* **41**, 459–468 (2009).

7. C. Rosin, J. R. Poulsen, V. Swamy, A. Granados, A pantropical assessment of vertebrate physical damage to forest seedlings and the effects of defaunation. *Glob. Ecol. Conserv.* **11**, 188–195 (2017).

8. A. W. Cardoso, Y. Malhi, I. Oliveras, D. Lehmann, J. E. Ndong, E. Dimoto, E. Bush, K. Jeffery, N. Labriere, S. L. Lewis, L. T. J. White, W. Bond, K. Abernethy, The role of forest elephants in shaping tropical forest–savanna coexistence. *Ecosystems* **23**, 602–616 (2020).

9. F. Berzaghi, F. Bretagnolle, C. Durand-Bessart, S. Blake, Megaherbivores modify forest structure and increase carbon stocks through multiple pathways. *Proc. Natl. Acad. Sci. U.S.A.* **120**, e2201832120 (2023).

10. F. Babweteera, P. Savill, N. Brown, *Balanites wilsoniana*: Regeneration with and without elephants. *Biol. Conserv.* **134**, 40–47 (2007).

11. A. Campos-Arceiz, S. Blake, Megagardeners of the forest – The role of elephants in seed dispersal. *Acta Oecol.* **37**, 542–553 (2011).

12. K. A. Abernethy, L. Coad, G. Taylor, M. E. Lee, F. Maisels, Extent and ecological consequences of hunting in Central African rainforests in the twenty-first century. *Philos. Trans. R. Soc. Lond B Biol. Sci.* **368**, 20120303 (2013).

13. F. Berzaghi, M. Longo, P. Ciais, S. Blake, F. Bretagnolle, S. Vieira, M. Scaranello, G. Scarascia-Mugnozza, C. E. Doughty, Carbon stocks in central African forests enhanced by elephant disturbance. *Nat. Geosci.* **12**, 725–729 (2019).

14. D. Beaune, B. Fruth, L. Bollache, G. Hohmann, F. Bretagnolle, Doom of the elephant-dependent trees in a Congo tropical forest. *For. Ecol. Manage.* **295**, 109–117 (2013).

15. W. D. Hawthorne, M. P. E. Parren, How important are forest elephants to the survival of woody plant species in Upper Guinean forests? *J. Trop. Ecol.* **16**, 133–150 (2000).

16. A. C. Nchanji, A. J. Plumptre, Seed germination and early seedling establishment of some elephant-dispersed species in Banyang-Mbo Wildlife Sanctuary, south-western Cameroon. *J. Trop. Ecol.* **19**, 229–237 (2003).

17. L. F. Fuzessy, T. G. Cornelissen, C. Janson, F. A. O. Silveira, How do primates affect seed germination? A meta-analysis of gut passage effects on neotropical plants. *Oikos* **125**, 1069–1080 (2016).

18. J. H. Connell, "On the role of natural enemies in preventing competitive exclusion in some marine animals and rain forest trees" in *Dynamics of Population* (Centre for Agricultural Publishing and Documentation, 1971) pp. 298–312.

19. D. H. Janzen, Herbivores and the number of tree species in tropical forests. *Am. Nat.* **104**, 501–528 (1970).

20. P. B. Adler, D. Smull, K. H. Beard, R. T. Choi, T. Furniss, A. Kulmatiski, J. M. Meiners, A. T. Tredennick, K. E. Veblen, Competition and coexistence in plant communities: Intraspecific competition is stronger than interspecific competition. *Ecol. Lett.* **21**, 1319–1329 (2018).

21. J. Terborgh, Enemies maintain hyperdiverse tropical forests. *Am. Nat.* **179**, 303–314 (2012).

22. R. Bagchi, R. E. Gallery, S. Gripenberg, S. J. Gurr, L. Narayan, C. E. Addis, R. P. Freckleton, O. T. Lewis, Pathogens and insect herbivores drive rainforest plant diversity and composition. *Nature* **506**, 85–88 (2014).

23. X. Song, J. Y. Lim, J. Yang, M. S. Luskin, When do Janzen-Connell effects matter? A phylogenetic meta-analysis of conspecific negative distance and density dependence experiments. *Ecol. Lett.* **24**, 608–620 (2021).

24. E. Lebrija-Trejos, P. B. Reich, A. Hernández, S. J. Wright, Species with greater seed mass are more tolerant of conspecific neighbours: A key driver of early survival and future abundances in a tropical forest. *Ecol. Lett.* **19**, 1071–1080 (2016).

25. E. P. Cochrane, The need to be eaten: *Balanites wilsoniana* with and without elephant seed-dispersal. *J. Trop. Ecol.* **19**, 579–589 (2003).

26. N. Sekar, X. Giam, N. P. Sharma, R. Sukumar, How much *Dillenia indica* seed predation occurs from Asian elephant dung? *Acta Oecol.* **70**, 53–59 (2016).

27. K. R. McConkey, Influence of faeces on seed removal from gibbon droppings in a dipterocarp forest in Central Borneo. *J. Trop. Ecol.* **21**, 117–120 (2005).

28. F. Magliocca, S. Quérouil, A. Gautier-Hion, Seed eating in elephant dung by two large mammals in the Congo Republic. *Revue d'écologie* **58**, 143–149 (2003).

29. V. Deblauwe, Life history, uses, trade and management of *Diospyros crassiflora* Hiern, the ebony tree of the Central African forests: A state of knowledge. *For. Ecol. Manag.* **481**, 118655 (2021).

30. G. E. Schatz, P. P. Lowry II, J.-M. Onana, T. Stévart, V. Deblauwe, "*Diospyros crassiflora*. The IUCN Red List of Threatened Species 2019" (e.T33048A2831968, IUCN, 2019).

31. S. Blake, "The Ecology of Forest Elephant Distribution and its Implications for Conservation," thesis, University of Edinburgh (2002).

32. C. Beirne, A. C. Meier, G. Brumagin, L. Jasperse-Sjolander, M. Lewis, J. Masseloux, K. Myers, M. Fay, J. Okouyi, L. J. T. White, J. R. Poulsen, Climatic and resource determinants of forest elephant movements. *Front. Ecol. Evol.* **8**, 96 (2020).

33. F. Feer, Morphology of fruits dispersed by African forest elephants. *Afr. J. Ecol.* **33**, 279–284 (1995).

34. P. R. Guimarães Jr., M. Galetti, P. Jordano, Seed dispersal anachronisms: Rethinking the fruits extinct megafauna ate. *PLOS ONE* **3**, e1745 (2008).

35. C. E. G. Tutin, R. J. Parnell, F. White, Protecting seeds from primates: Examples from *Diospyros* spp. in the Lopé Reserve, Gabon. *J. Trop. Ecol.* **12**, 371–384 (1996).

36. K. R. McConkey, H. S. Sushma, A. Sengupta, Seed dispersal by frugivores without seed swallowing: Evaluating the contributions of stomatochoric seed dispersers. *Funct. Ecol.* **38**, 480–499 (2024).

37. J. Refisch, I. Koné, *Influence du braconnage sur les populations simiennes et effets secondaires sur la végétation: Un exemple tiré d'une région forestière de régime pluvieux en Côte d'Ivoire* (Deutsche Gesellschaft für Technische Zusammenarbeit, 2001). [Influence of poaching on simian populations and secondary effects on vegetation: An example from a forest region with a pluvial climate in Ivory Coast].

38. L. J. T. White, "Vegetation history and logging disturbance: Effects on rain forest in the Lope Reserve, Gabon (with special emphasis on elephants and apes)," thesis, University of Edinburgh (1992).

39. B. Dehaudt, T. Bruce, V. Deblauwe, A. Ferraz, B. Gardner, T. G. B. Bibila, M. LeBreton, G. Mempong, K. Njabo, S. N. Nkengbeza, E. M. Ordway, L. Pavan, N. J. Russo, T. B. Smith,

M. S. Luskin, Divergent seed dispersal outcomes: Interactions between seed, disperser, and forest traits. *Ecology* **105**, e4409 (2024).

40. M. Delibes, I. Castañeda, J. M. Fedriani, Spitting seeds from the cud: A review of an endozoochory exclusive to ruminants. *Front. Ecol. Evol.* **7**, 265 (2019).

41. R. K. Kobe, C. F. Vriesendorp, Conspecific density dependence in seedlings varies with species shade tolerance in a wet tropical forest. *Ecol. Lett.* **14**, 503–510 (2011).

42. I. Abiem, I. Dickie, D. Kenfack, H. Chapman, Conspecific negative density dependence does not explain coexistence in a tropical Afromontane forest. *J. Veg. Sci.* **32**, e12990 (2021).

43. T. G. Seidler, J. B. Plotkin, Seed dispersal and spatial pattern in tropical trees. *PLOS Biol.* **4**, e344 (2006).

44. X. Vekemans, O. J. Hardy, New insights from fine-scale spatial genetic structure analyses in plant populations. *Mol. Ecol.* **13**, 921–935 (2004).

45. C. W. Dick, O. J. Hardy, F. A. Jones, R. J. Petit, Spatial scales of pollen and seed-mediated gene flow in tropical rain forest trees. *Trop. Plant Biol.* **1**, 20–33 (2008).

46. J. R. Poulsen, C. Beirne, C. Rundel, M. Baldino, S. Kim, J. Knorr, T. Minich, L. Jin, C. L. Núñez, S. Xiao, W. Mbamy, G. N. Obiang, J. Masseloux, T. Nkoghe, M. O. Ebanega, C. J. Clark, M. J. Fay, P. Morkel, J. Okouyi, L. J. T. White, J. P. Wright, Long distance seed dispersal by forest elephants. *Front. Ecol. Evol.* **9**, 789264 (2021).

47. P. A. Jansen, B. T. Hirsch, W.-J. Emsens, V. Zamora-Gutierrez, M. Wikelski, R. Kays, Thieving rodents as substitute dispersers of megafaunal seeds. *Proc. Natl. Acad. Sci. U.S.A.* **109**, 12610–12615 (2012).

48. O. Bhasin, J.-L. Doucet, R. Ndonda Makemba, J.-F. Gillet, V. Deblauwe, B. Sonké, O. J. Hardy, Contrasted spatial, demographic and genetic structures of a light-demanding African timber species, *Cylicodiscus gabunensis* Harms – Implications for a sustainable management of its populations. *For. Ecol. Manag.* **551**, 121527 (2024).

49. D. M. A. Angbonda, F. K. Monthe, N. Bourland, F. Boyemba, O. J. Hardy, Seed and pollen dispersal and fine-scale spatial genetic structure of a threatened tree species: *Pericopsis elata* (HARMS) Meeuwen (Fabaceae). *Tree Genet. Genom.* **17**, 27 (2021).

50. O. J. Hardy, B. Delaide, H. Hainaut, J.-F. Gillet, P. Gillet, E. Kaymak, N. Vankerckhove, J. Duminil, J.-L. Doucet, Seed and pollen dispersal distances in two African legume timber trees and their reproductive potential under selective logging. *Mol. Ecol.* **28**, 3119–3134 (2019).

51. V. Droissart, L. Azandi, E. R. Onguene, M. Savignac, T. B. Smith, V. Deblauwe, PICT: A low-cost, modular, open-source camera trap system to study plant–insect interactions. *Methods Ecol. Evol.* **12**, 1389–1396 (2021).

52. B. C. Wang, V. L. Sork, M. T. Leong, T. B. Smith, Hunting of mammals reduces seed removal and dispersal of the afrotropical tree *Antrocaryon klaineanum* (Anacaridaceae). *Biotropica* **39**, 340–347 (2007).

53. C. Rosin, J. R. Poulsen, Hunting-induced defaunation drives increased seed predation and decreased seedling establishment of commercially important tree species in an Afrotropical forest. *For. Ecol. Manag.* **382**, 206–213 (2016).

54. P. J. Williams, R. C. Ong, J. F. Brodie, M. S. Luskin, Fungi and insects compensate for lost vertebrate seed predation in an experimentally defaunated tropical forest. *Nat. Commun.* **12**, 1650 (2021).

55. R. Dirzo, E. Mendoza, P. Ortíz, Size-related differential seed predation in a heavily defaunated neotropical rain forest. *Biotropica* **39**, 355–362 (2007).

56. L. Pavan, "Caught in the snare: The indirect effects of hunting-induced mammal defauntion on bird communities," thesis, Stanford University (2022).

57. S. P. Hubbell, J. A. Ahumada, R. Condit, R. B. Foster, Local neighborhood effects on long-term survival of individual trees in a neotropical forest. *Ecol. Res.* **16**, 859–875 (2001).

58. Y. Malhi, C. Girardin, D. B. Metcalfe, C. E. Doughty, L. E. O. C. Aragão, S. W. Rifai, I. Oliveras, A. Shenkin, J. Aguirre-Gutiérrez, C. A. Dahlsjö, T. Riutta, E. Berenguer, S. Moore, W. H. Huasco, N. Salinas, A. C. L. da Costa, L. P. Bentley, S. Adu-Bredu, T. R. Marthews, P. Meir, O. L. Phillips, The Global Ecosystems Monitoring network: Monitoring ecosystem productivity and carbon cycling across the tropics. *Biol. Conserv.* **253**, 108889 (2021).

59. E. R. Bush, R. C. Whytock, L. Bahaa-el-din, S. Bourgeois, N. Bunnefeld, A. W. Cardoso, J. T. Dikangadissi, P. Dimbonda, E. Dimoto, J. E. Ndong, K. J. Jeffery, D. Lehmann, L. Makaga, B. Momboua, L. R. W. Momont, C. E. G. Tutin, L. J. T. White, A. Whittaker, K. Abernethy, Long-term collapse in fruit availability threatens Central African forest megafauna. *Science* **370**, 1219–1222 (2020).

60. N. J. Russo, A. B. Davies, R. V. Blakey, E. M. Ordway, T. B. Smith, Feedback loops between 3D vegetation structure and ecological functions of animals. *Ecol. Lett.* **26**, 1597–1613 (2023).

61. C. E. Doughty, A. Wolf, N. Morueta-Holme, P. M. Jørgensen, B. Sandel, C. Violle, B. Boyle, N. J. B. Kraft, R. K. Peet, B. J. Enquist, J.-C. Svenning, S. Blake, M. Galetti, Megafauna extinction, tree species range reduction, and carbon storage in Amazonian forests. *Ecography* **39**, 194–203 (2016).

62. Y. Malhi, C. E. Doughty, M. Galetti, F. A. Smith, J.-C. Svenning, J. W. Terborgh, Megafauna and ecosystem function from the Pleistocene to the Anthropocene. *Proc. Natl. Acad. Sci. U.S.A.* **113**, 838–846 (2016).

63. C. Rosin, J. R. Poulsen, Telemetric tracking of scatterhoarding and seed fate in a Central African forest. *Biotropica* **49**, 170–176 (2017).

64. A. M. Osuri, J. Ratnam, V. Varma, P. Alvarez-Loayza, J. Hurtado Astaiza, M. Bradford, C. Fletcher, M. Ndoundou-Hockemba, P. A. Jansen, D. Kenfack, A. R. Marshall, B. R. Ramesh, F. Rovero, M. Sankaran, Contrasting effects of defaunation on aboveground carbon storage across the global tropics. *Nat. Commun.* **7**, 11351 (2016).

65. ESA. Land Cover CCI Product User Guide Version 2. Tech. Rep. (2017); maps.elie.ucl.ac.be/CCI/viewer/download/ESACCI-LC-Ph2-PUGv2_2.0.pdf.

66. T. Bruce, C. Ndjassi, A. Fowler, M. Ndimbe, O. Fankem, R. Tabue Mbobda, A.-S. Kobla, F. Wabo Puemo, R. Amin, T. Wacher, "Faunal inventory of the Dja Faunal Reserve, Cameroon" (2018).

67. O. Fankem, A.-S. Kobla, R. Tene, M. Ndimbe, H. Fopa, G. Oum Ndjock, R. Amin, A. Fowler, "Inventaire faunique de la Réserve de Faune du Dja, Cameroon - 2021" (Ministère Des Forêts Et de la Faune, Zoological Society of London, Global Conservation, African Wildlife Foundation, 2022).

68. F. Maisels, S. Strindberg, S. Blake, G. Wittemyer, J. Hart, E. A. Williamson, R. Aba'a, G. Abitsi, R. D. Ambahe, F. Amsini, P. C. Bakabana, T. C. Hicks, R. E. Bayogo, M. Bechem, R. L. Beyers, A. N. Bezangoye, P. Boundja, N. Bout, M. E. Akou, L. B. Bene, B. Fosso, E. Greengrass, F. Grossmann, C. Ikamba-Nkulu, O. Ilambu, B.-I. Inogwabini, F. Iyenguet, F. Kiminou, M. Kokangoye, D. Kujirakwinja, S. Latour, I. Liengola, Q. Mackaya, J. Madidi, B. Madzoke, C. Makoumbou, G.-A. Malanda, R. Malonga, O. Mbani, V. A. Mbendzo, E. Ambassa, A. Ekinde, Y. Mihindou, B. J. Morgan, P. Motsaba, G. Moukala, A. Mounguengui, B. S. Mowawa, C. Ndzai, S. Nixon, P. Nkumu, F. Nzolani, L. Pintea, A. Plumptre, H. Rainey, B. B. de Semboli, A. Serckx, E. Stokes, A. Turkalo, H. Vanleeuwe, A. Vosper, Y. Warren, Devastating decline of forest elephants in Central Africa. *PLOS ONE* **8**, e59469 (2013).

69. J. R. Poulsen, S. E. Koerner, S. Moore, V. R. Medjibe, S. Blake, C. J. Clark, M. E. Akou, M. Fay, A. Meier, J. Okouyi, C. Rosin, L. J. T. White, Poaching empties critical Central African wilderness of forest elephants. *Curr. Biol.* **27**, R134–R135 (2017).

70. C. Kengne Olivier, L. Zapfack, C. Garcia, V. Noiha Noumi, B.-A. Nkongmeneck, Diversité floristique et structurale de deux forêts communautaires sous exploitation au Cameroun: cas de Kompia et Nkolenyeng. *Eur. Sci. J.* **14**, 245 (2018). [Floristic and structural diversity of two community forests under logging in Cameroon: the case of Kompia and Nkolenyeng].

71. BUCREP, "Répertoire actualisé des villages du Cameroun: Troisième Recensement Général de la Population et de l'Habitat du Cameroun" (BUCREP, 2005). [Updated directory of villages of Cameroon: third general census of population and housing of Cameroon].

72. ORSTOM, *Dictionnaire des villages du département du Haut Nyong*, vol. 15, *Répertoire Géographique du Cameroun* (ORSTOM, 1968), pp. XI-67. [Dictionary of the villages of the Haut-Nyong department].

73. W. Delvingt, M. Dethier, P. Auzel, P. Jeanmart, "La chasse villageoise Badjoué, gestion coutumière durable ou pillage de la ressource gibier?" in *La foret des hommes: Terroirs villageois en foret tropicale africaine*, W. Delvingt, Ed. (Presses Agronomiques de Gembloux, 2001), chap. 4, pp. 65–92. ["Village hunting among the Badjoué: sustainable customary management or plundering of the game resource?" in The forest of people: village territories in the African tropical forest].

74. J. Morellet, Une expérience sylvicole au Cameroun. *Bois et Forêts des Tropiques* **25**, 297–312 (1952). [A silvicultural experiment in Cameroon].

75. ORSTOM, *Dictionnaire des villages du Nyong et Soo,* vol. 5, *Repertoire Oceanographique du Cameroun* (ORSTOM, 1965), pp. IV-31. [Dictionary of the villages of the Nyong and Soo department].

76. J. C. Mey Boudoug, S. Dewang Dyio, C. N. S. Fotie, J. Etah Kang, I. Ndzana, V. R. Nguimdo Vouffo, M. M. Mbolo, Preliminary evidence of Great Apes' occurrence in peri-urban and highly degraded forest reserves in Cameroon. *Afr. J. Ecol.* **63**, e70022 (2025).

77. A. Baddeley, E. Rubak, R. Turner, *Spatial Point Patterns: Methodology and Applications with R*, Interdisciplinary Statistics Series, CRC Press, 2015).

78. A. J. Baddeley, J. Møller, R. Waagepetersen, Non- and semi-parametric estimation of interaction in inhomogeneous point patterns. *Stat. Neerl.* **54**, 329–350 (2000).

79. A. Baddeley, R. Turner, spatstat: An R package for analyzing spatial point patterns. *J. Stat. Softw.* **12**, 1–42 (2005).

80. C. Micheneau, G. Dauby, N. Bourland, J.-L. Doucet, O. J. Hardy, Development and characterization of microsatellite loci in *Pericopsis elata* (Fabaceae) using a cost-efficient approach. *Am. J. Bot.* **98**, e268–e270 (2011).

81. M. Schuelke, An economic method for the fluorescent labeling of PCR fragments. *Nat. Biotechnol.* **18**, 233–234 (2000).

82. O. J. Hardy, X. Vekemans, SPAGEDI: A versatile computer program to analyse spatial genetic structure at the individual or population levels. *Mol. Ecol. Notes* **2**, 618–620 (2002).

83. B. A. Loiselle, V. L. Sork, J. Nason, C. Graham, Spatial genetic structure of a tropical understory shrub, *Psychotria officinalis* (Rubiaceae). *Am. J. Bot.* **82**, 1420–1425 (1995).

84. I. J. Chybicki, NMπ—Improved re-implementation of NM+, a software for estimating gene dispersal and mating patterns. *Mol. Ecol. Resour.* **18**, 159–168 (2018).

85. S. Nakagawa, H. Schielzeth, A general and simple method for obtaining R2 from generalized linear mixed-effects models. *Methods Ecol. Evol.* **4**, 133–142 (2013).

86. IUCN SSC African Elephant Specialist Group, "*Loxodonta cyclotis* (spatial data). The IUCN Red List of Threatened Species 2021" (2025); www.iucnredlist.org.

87. H. S. Grantham, A. Duncan, T. D. Evans, K. R. Jones, H. L. Beyer, R. Schuster, J. Walston, J. C. Ray, J. G. Robinson, M. Callow, T. Clements, H. M. Costa, A. DeGemmis, P. R. Elsen, J. Ervin, P. Franco, E. Goldman, S. Goetz, A. Hansen, E. Hofsvang, P. Jantz, S. Jupiter, A. Kang, P. Langhammer, W. F. Laurance, S. Lieberman, M. Linkie, Y. Malhi, S. Maxwell, M. Mendez, R. Mittermeier, N. J. Murray, H. Possingham, J. Radachowsky, S. Saatchi, C. Samper, J. Silverman, A. Shapiro, B. Strassburg, T. Stevens, E. Stokes, R. Taylor, T. Tear, R. Tizard, O. Venter, P. Visconti, S. Wang, J. E. M. Watson, Anthropogenic modification of forests means only 40% of remaining forests have high ecosystem integrity. *Nat. Commun.* **11**, 5978 (2020).

88. R. Letouzey, *Notice de la carte phytogéographique du Cameroun au 1/500000* (Institut de la carte internationale de la végétation, 1985), p. 240. [Explanatory note to the phytogeographic map of Cameroon at 1:500000].

89. D. N. Karger, O. Conrad, J. Böhner, T. Kawohl, H. Kreft, R. W. Soria-Auza, N. E. Zimmermann, H. P. Linder, M. Kessler, Climatologies at high resolution for the earth's land surface areas. *Sci. Data* **4**, 170122 (2017).

90. T. M. Culley, S. G. Weller, A. K. Sakai, K. A. Putnam, Characterization of microsatellite loci in the Hawaiian endemic shrub *Schiedea adamantis* (Caryophyllaceae) and amplification in related species and genera. *Mol. Ecol. Resour.* **8**, 1081–1084 (2008).
